# CO_2_-Assisted asymmetric hydrogenation of prochiral allylamines†

**DOI:** 10.1039/d2ra00263a

**Published:** 2022-02-28

**Authors:** Tamara M. de Winter, Jaddie Ho, Christopher J. Alridge, Philip G. Jessop

**Affiliations:** Department of Chemistry, Queen's University Kingston ON K7L 3N6 Canada jessop@queensu.ca

## Abstract

A new methodology for the asymmetric hydrogenation of allylamines takes advantage of a reversible reaction between amines and carbon dioxide (CO_2_) to suppress unwanted side reactions. The effects of various parameters (pressure, time, solvent, and base additives) on the enantioselectivity and conversion of the reaction were studied. The homogeneously-catalyzed asymmetric hydrogenation of 2-arylprop-2-en-1-amine resulted in complete conversion and up to 82% enantiomeric excess (ee). Added base, if chosen carefully, improves the enantioselectivity and chemoselectivity of the overall reaction.

## Abstract 2

CO_2_ can act as an *in situ* protecting group for the asymmetric hydrogenation of allylamines.

## Graphical abstract

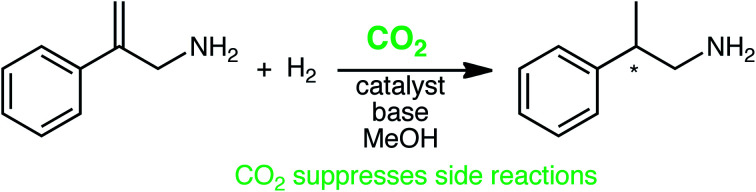

## Introduction

Optically active amines are used as pharmaceuticals, agrochemicals and resolving agents or chiral auxiliaries.^1–6^ Many efforts have been directed towards the enantioselective hydrogenation of C=C and C=N double bonds for the synthesis of chiral amines.^1,3,4,7–13^

**Scheme 1:**
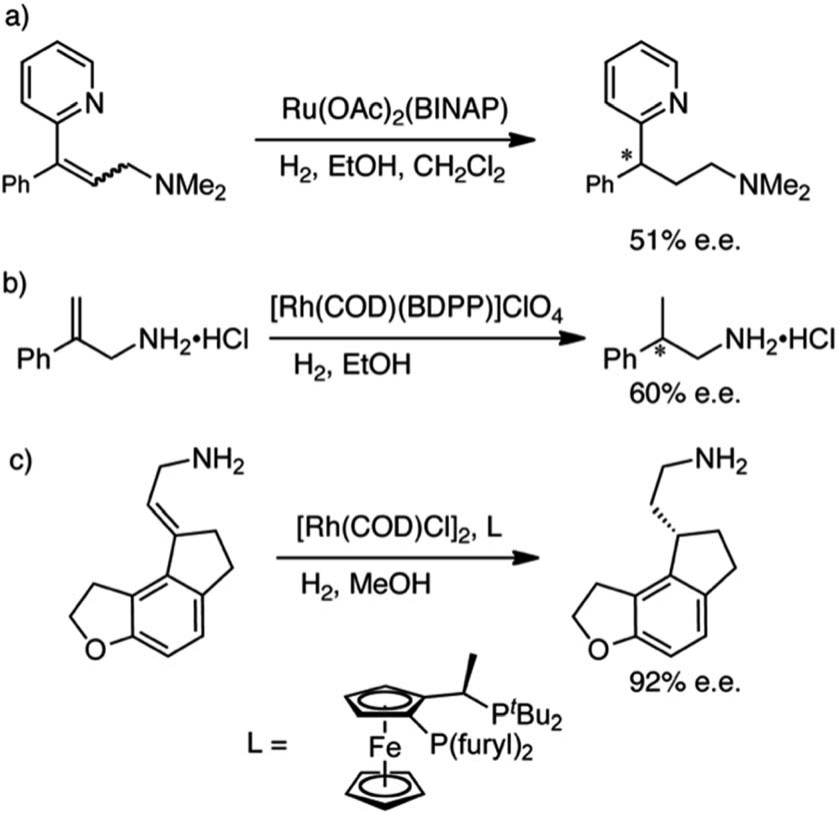
Literature examples of the hydrogenation of allyamines. (a) Botteghi *et al.*^14^ (b) Fahrang *et al.*^15^ (c) Yamashita and Yamano.^16^

We sought an asymmetric hydrogenation of prochiral allylamines, in the expectation that it would represent a more direct, efficient and greener synthesis of chiral amines than current asymmetric hydrogenations of *N*-protected allylamines. Until today, the direct hydrogenation of unprotected allylamines has been largely ignored; the few examples are shown in Scheme 1. Botteghi *et al.*^14^ reported that the hydrogenation gave low yields due to the unwanted hydrogenolysis of the C–N bond. Fahrang *et al.*^15^ strategically hydrogenated the hydrogen chloride salt of their allylamine but did not comment on yield or purity of the product. However, both groups reported only moderate enantioselectivity. Yamashita and Yamano^16^ screened multiple Josiphos ligands to find one with good enantioselectivity for the hydrogenation of a precursor of Ramelteon, a melatonin receptor agonist.

**Scheme 2:**
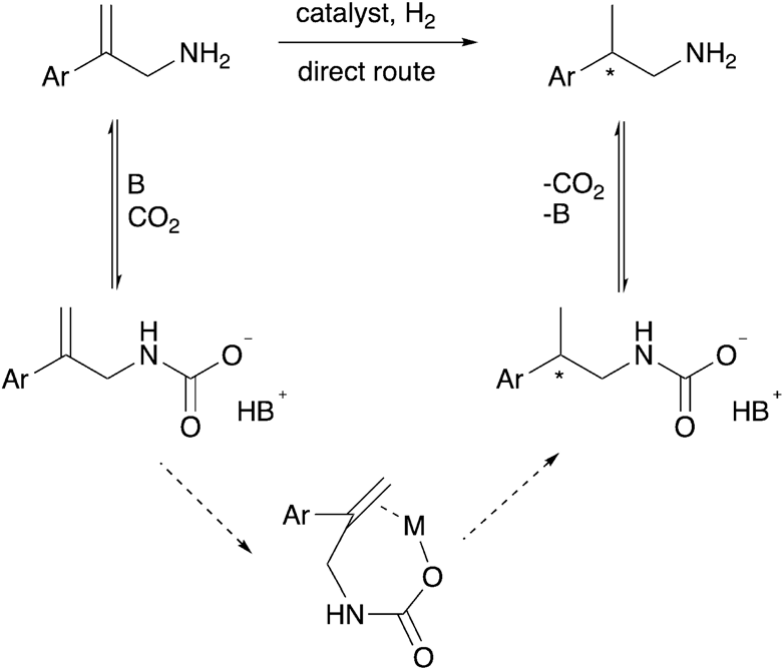
Upper route with solid arrows: the yield of the direct hydrogenation could be increased by the starting allylamine and/or the product being stabilized as the carbamate, even though the hydrogenation step itself involves the allylamine rather than the carbamate. Lower route with dotted arrows: alternatively, the yield and enantioselectivity could both be improved by the allylcarbamate binding to the metal centre, allowing chelation during the hydrogen transfer step.

We hypothesized that CO_2_ could act as an *in situ* protecting group in a way that protects the amine from undesired side reactions. This could potentially replace the *N*-acetyl protecting group that is currently used for asymmetric hydrogenation of protected allylamines. The CO_2_ would reversibly convert the allylamine substrate and/or the chiral amine product into a carbamate or carbamic acid (solid arrows in Scheme 2), which would circumvent additional steps of amine protection and deprotection, ultimately giving a more economical synthesis. In separate reports by Chatterjee *et al.*^17^ and Xie *et al.*^18^ CO_2_ was used in the hydrogenation of nitriles and imines to protect the desired amine products, by the formation of a carbamic acid, from undergoing undesired further reactions. Thus the carbamic acid acts as an *in situ* protecting group for the kinetic product during the hydrogenation.^17,18^ Fortunately, the reaction of allylamines with CO_2_ to form carbamic acids or carbamate anions is known, but in the context of synthesizing cyclic carbamate esters.^19–25^

In addition to serving as a protecting group, the carbamic acid or carbamate anion produced by the reaction of CO_2_ with the amine might serve as a better metal-binding functional group (lower part of Scheme 2), allowing chelation in the hydrogenation transition state as occurs when unsaturated carboxylic acids are asymmetrically hydrogenated.^26^ The olefin binding step in the catalytic cycle, in which the C=C double bond is bound to the metal centre prior to insertion into the M–H bond, would thereby become an intramolecular rather than intermolecular step, which would quite feasibly enhance enantioselectivity.

To explore these two intriguing hypotheses, we chose to study the asymmetric hydrogenation of a primary allylamine with and without CO_2_, and with and without added base. The option of adding a base was included in order to promote the formation of carbamate anions rather than carbamic acids.

## Results and discussion

**Scheme 3:**
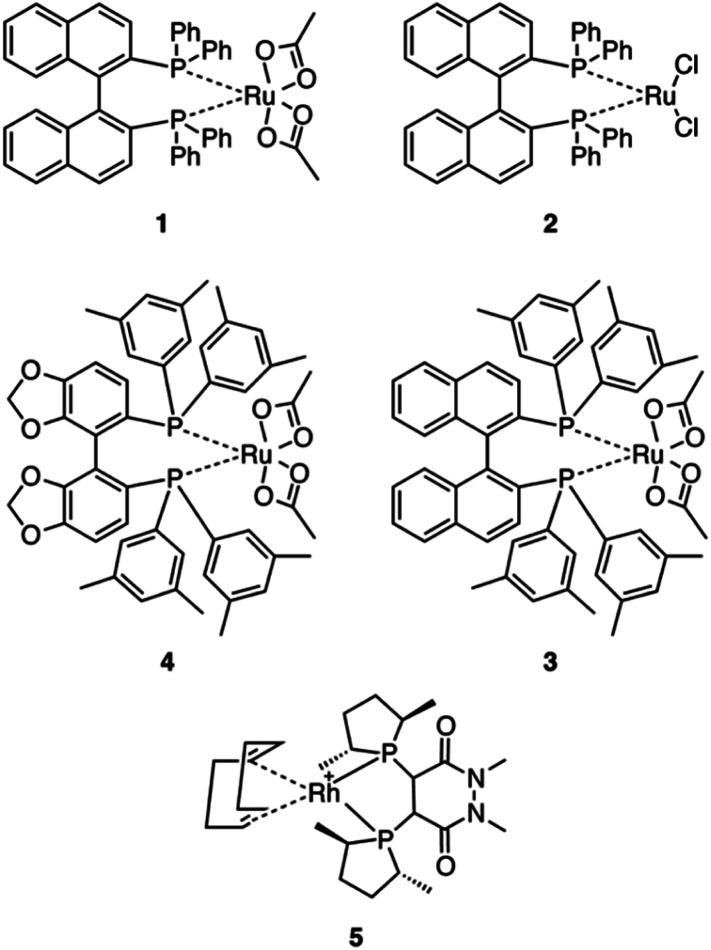
The catalysts initially tested for the asymmetric hydrogenation of prochiral allylamines.

Five commercially available catalysts (Scheme 3) were chosen based upon their ability to asymmetrically hydrogenate prochiral unsaturated carboxylic acids. The catalysts were diacetato[(*R*)-(+)-2,2′-bis(diphenylphosphino)-1,1′-binaphthyl]ruthenium(ii), 1,^26^ dichloro[(*S*)-(−)-2,2′-bis(diphenylphosphino)-1,1′-binaphthyl]ruthenium(ii), 2, diacetato{(*R*)-(+)-2,2′-bis[di(3,5-xylyl)phosphino]-1,1′-binaphthyl}ruthenium(ii), 3, diacetato{(*R*)-(+)-5,5′-bis[di(3,5-xylyl)phosphino]-4,4′-bi-1,3-benzodioxole} ruthenium(ii), 4,^27^ and (−)-4,5-bis[(2*R*,5*R*)-2,5-dimethylphospholanyl](1,2-dimethyl-1,2′-dihydropyridazine-3,6-dione)(1,5-cyclooctadiene)rhodium(i) tetrafluoroborate, 5.^10^

**Scheme 4:**
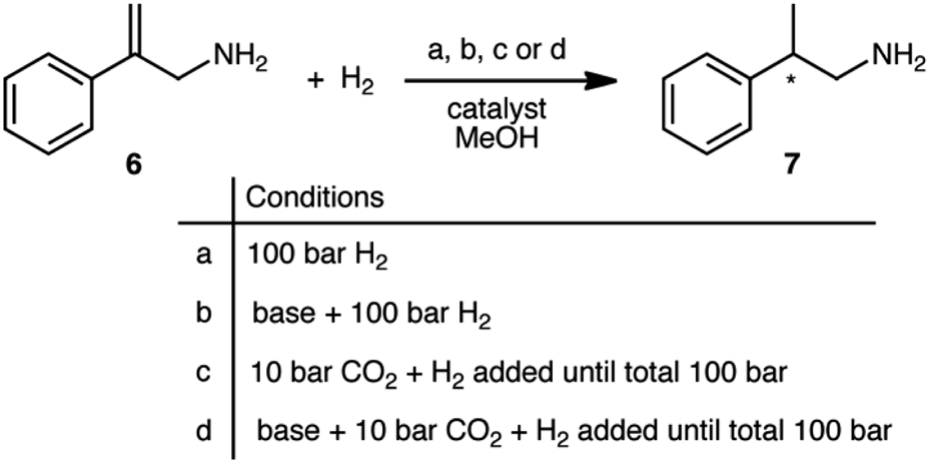
The four reaction conditions used for the asymmetric hydrogenation scheme of 2-phenylprop-2-en-1-amine, 6.

The prochiral allylamine substrate that was chosen for initial screening of catalysts and conditions was 2-phenylprop-2-en-1-amine, 6 (Scheme 4). Four hydrogenation conditions were investigated; each catalyst listed above was tested with (a) only H_2_, (b) H_2_ and base, (c) H_2_ and CO_2_, and lastly (d) H_2_, CO_2(g)_ and base. 1,8-Diazabicyclo[5.4.0]undec-7-ene (DBU) was the base chosen as it is more basic than the allylic amine. Initial experiments were conducted at 24 h reaction time in order to maximize the chances that at least one catalyst would be able to give a significant yield.

The results show that the conditions greatly affect the asymmetric hydrogenation of 6 (Table 1, 24 h). With solely H_2_, all catalysts produced 2-phenylpropan-1-amine, 7, in low to moderate yields and enantioselectivity (ee), consistent with the findings of Botteghi *et al.*^14^ Catalyst 1 gave the highest yield, 72%, and catalyst 5 gave the best ee, 74%.

**Table 1:** Asymmetric hydrogenation of 2-phenylprop-2-en-1-amine, 6, under 100 bar total pressure with and without CO_2(g)_ and DBU^a^

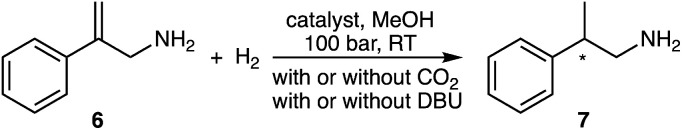			
Additive	Cat.	% Yield (% ee)	
		24 h	14–15 h
None^b^	1	72 (33)	79 (33)
	2	32 (30)	72 (31)
	3	35 (48)	66 (39)
	4	38 (60)	67 (57)
	5	29 (74)	57 (68)
DBU^c^	1	21 (50)	48 (45)
	2	38 (52)	60 (46)
	3	38 (49)	52 (42)
	4	48 (49)	58 (48)
	5	54 (31)	50 (26)
CO_2_^d^	1	64 (26)	70 (23)
	2	85 (26)	72 (25)
	3	45 (36)	82 (36)
	4	56 (41)	84 (37)
	5	64 (77)	84 (75)
CO_2_ + DBU^e^	1	68 (25)	73 (25)
	2	96 (31)	92 (25)
	3	64 (31)	69 (36)
	4	50 (40)	62 (49)
	5	96 (75)	94 (73)

a Experiments were done in triplicate and at RT in a 160 mL stainless steel vessel containing 10 mg 6 and 2 mL methanol in a 1 dram vial under 100 bar total pressure. Conversions for all reactions above were >95% and the experimental error for % yield and % ee were ±10 and ±4, respectively. Catalysts 2 and 5 produced (*S*)-7, while catalysts 1, 3, and 4 produced (*R*)-7. Yields are ^1^H NMR values measured with an internal standard (1,3,5-trimethoxybenzene). Enantiomeric excess was determined by HPLC.

b 100 bar H_2_.

c 100 bar H_2_, 1 eq. DBU added (relative to 6).

d 10 bar CO_2(g)_ added, followed by enough H_2(g)_ to bring the total pressure to 100 bar.

e 10 bar CO_2(g)_ added, followed by enough H_2(g)_ to bring the total pressure to 100 bar, 1 eq. DBU added (relative to 6).

The addition of DBU made minor improvements to the enantioselectivity of catalysts 1 and 2, but was otherwise unhelpful. The addition of CO_2_ without base increased the yield (Table 1) and the purity of the product (by suppressing side products, Fig. S4†) but not the enantioselectivity for catalysts 2–5. The addition of CO_2_ with base dramatically increased the yield with all catalysts except 4. The enantioselectivity of the hydrogenation was adversely affected for all catalysts except 5. The best result, with high yield (96%) and reasonable ee (75%), was obtained with catalyst 5 in the presence of both CO_2_ and base.

Following the positive results for the asymmetric hydrogenation at 24 h, the reaction time was investigated (Table 1, 14–15 h). With a decreased reaction time of 14–15, an increase in yield was observed for almost all catalysts and conditions, suggesting that extended reaction times allow the desired products to undergo further reactions giving unwanted products. However, enantioselectivities were not significantly changed by the decrease in reaction time. Even shorter reaction times give lower enantioselectivity (Table S2†).

The success of catalyst 5 suggests that Rh-based catalysts may be more suitable than classical Ru BINAP catalysts for the asymmetric hydrogenation of 2-phenylprop-2-en-1-amine, 6. This seems surprising if one considers allylamines to be close analogues of allylic alcohols, for which the classical Ru BINAP catalysts are known to be excellent hydrogenation catalysts.^28^ However, perhaps a better analogy would be to the β-ketoamines, for which cationic Rh complexes are better hydrogenation catalysts than the classical Ru BINAP complexes.^29^ In the proposed transition state for those hydrogenations, the amine group binds to the Rh centre and the C=O double bond then undergoes Rh–H insertion leading to hydrogenation.^29^ A similar mechanism may operate for the asymmetric hydrogenation of allylamines, although it is worth noting that a DFT study of the mechanism for asymmetric isomerization of allylamines (for which Rh catalysts are again superior to Ru) shows that the nitrogen is not coordinated during the hydrogen transfer step.^30^

**Scheme 5:**
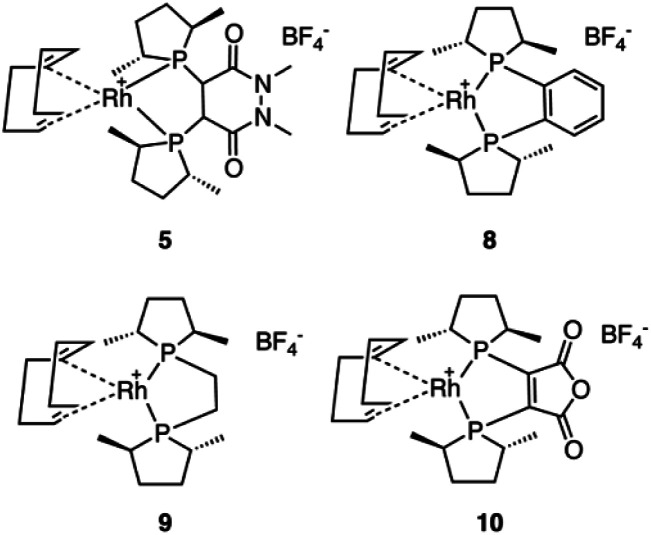
The four rhodium(i) catASium® catalysts applied to the asymmetric hydrogenation of 6.

In light of the success of catalyst 5, three more Rh based catalysts were chosen from the catASium® family (Scheme 5) with the corresponding ligands (*R*,*R*)-Me-DUPHOS, 8, (*R*,*R*)-Me-BPE-Rh, 9,^8^ and 3,4-bis-[(*R*,*R*)-(2,5-dimethylphospholan-1-yl]maleic anhydride 10.^8,31^ Unfortunately, compared to catalyst 5, catalysts 8, 9 and 10 did not provide improved results, with catalyst 10 yielding similar results (Table S1†).

H_2_ pressure is known to affect hydrogenation enantioselectivity.^26^ To evaluate the effect of H_2_ pressure in the present system, a lower pressure was tested. The reaction time was increased to 24 h to compensate for the anticipated lower rate of reaction. Unfortunately, the lowered H_2_ pressure decreased the performance of catalyst 5 and caused no significant improvements with 8 and 9 (Table S1†).

Next, the effect of solvent on the reaction was examined (Table 2). For catalyst 8 it was reported that the best solvents for the asymmetric hydrogenations of α-aminomethylacrylates,^26^ ene-carbonates,^3^ β-acylamido acrylates,^3^ and enamides^4^ were isopropanol (IPA), methanol (MeOH), and tetrahydrofuran (THF). For this reason, the asymmetric hydrogenation of 6 in the presence of CO_2_ was tested in these solvents with catalysts 5 and 8 but no significant improvement was obtained relative to the results with catalyst 5 in MeOH.

**Table 2:** The effects of different solvents and auxiliary bases on the conversion and enantioselectivity of the asymmetric hydrogenation of 2-phenylprop-2-en-1-amine, 6 a

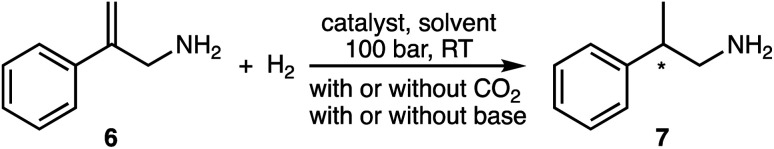				
Additive	Cat.	% Yield (% ee)		
		MeOH	IPA	THF
None^b^	5	57 (68)	65 (62)	52 (3)
	8	66 (61)	62 (64)	52 (7)
DBU^c^	5	50 (26)	73 (17)	69 (4)
	8	71 (69)	71 (25)	58 (1)
CyNMe_2_^c^	5	58 (64)	62 (44)	—
	8	—	60 (63)	—
iPr_2_NEt^c^	5	66 (59)	56 (64)	—
	8	—	48 (65)	—
CO_2_^d^	5	84 (75)	79 (60)	40^f^ (14)
	8	54 (65)	83 (69)	46 (43)
CO_2_ + DBU^e^	5	94 (73)	96 (50)	36^f^ (22)
	8	72 (69)	80 (70)	31^f^ (6)
	10	69 (76)	—	—
CO_2_ + CyNMe_2_^e^	5	77 (71)	80 (55)	—
	8	—	78 (72)	—
	10	95 (71)	—	—
CO_2_ + iPr_2_NEt^e^	5	>99 (69)	75 (72)	—
	8	—	83^g^ (72)	—
	10	85 (73)	—	—

a Experiments were done in triplicate and at RT in a 160 mL stainless steel vessel containing 10 mg 6 and 2 mL of the indicated solvent in a 1 dram vial under 100 bar total pressure. Reaction time was 14–15 h. Conversions for all reactions above were ≥93% and the experimental error for % yield and % ee were ±10 and ±4, respectively. Catalysts 5 and 8 produced (*S*)-7. Catalyst 10 produced (*R*)-7. Yields are ^1^H NMR values measured with an internal standard (1,3,5-trimethoxybenzene). Enantiomeric excess determined by HPLC.

b 100 bar H_2_.

c 100 bar H_2_, 1 eq. of base added (relative to 6).

d 10 bar CO_2(g)_ added, followed by enough H_2(g)_ to bring the total pressure to 100 bar.

e 10 bar CO_2(g)_ added, followed by enough H_2(g)_ to bring the total pressure to 100 bar, 1 eq. of base added (relative to 6).

f Conversion% 70–76%.

g Conversion% 83%.

**Scheme 6:**
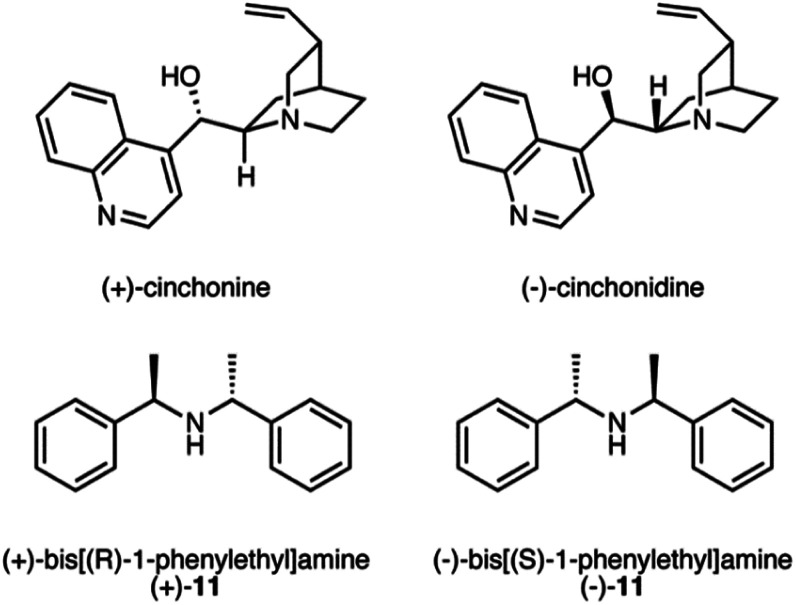
Chiral bases used in the asymmetric hydrogenation of 6.

Despite the complete conversion of 6 in all runs, yields were low in many instances and unidentified peaks were observed in the ^1^H NMR spectra. Even though the overall yields were found to be higher with the addition of DBU, we suspected that the use of DBU as the base may be leading to or assisting the decomposition of either the starting material or product. Therefore, we investigated the use of weaker bases (Table 2). *N*,*N*-Dimethylcyclohexylamine (CyNMe_2_), and *N*,*N*-diisopropylethylamine (iPr_2_NEt) were tested with catalysts 5 and 8 in the solvent that provided the best results for each (catalyst 5 with MeOH and IPA, and catalyst 8 with IPA). The best condition for catalyst 5 were found in the presence of H_2_, CO_2_, MeOH and DBU. However, CyNMe_2_ produced the cleanest reaction by ^1^H NMR spectroscopy with comparable enantioselectivity. For catalyst 8, the addition of CO_2_, IPA and iPr_2_NEt resulted in the highest yield (83%) and ee (72%). Catalyst 10 gave decent results in methanol with CO_2_ and the weaker bases, but the best overall result is still with catalyst 5 in the presence of CO_2_ and DBU. Using chiral bases (Scheme 6) caused modest improvements in the enantioselectivity with catalyst 8 but not catalyst 5 (Table S3†). The enantioselectivity was not affected by the chirality of the base, presumably because the chiral bases, in their cationic form, were not close enough to the catalytic centre to induce a chiral environment. Therefore, the success of these chiral bases at mildly improving the enantioselectivity is due to their weaker basicity rather than their chirality.

Using the best hydrogenation conditions (CO_2_ + DBU in MeOH for 24 h) with catalyst 5, the reaction was scaled to 650 mg to obtain an isolated yield of 416 mg (64%, Fig. S5†).

**Scheme 7:**
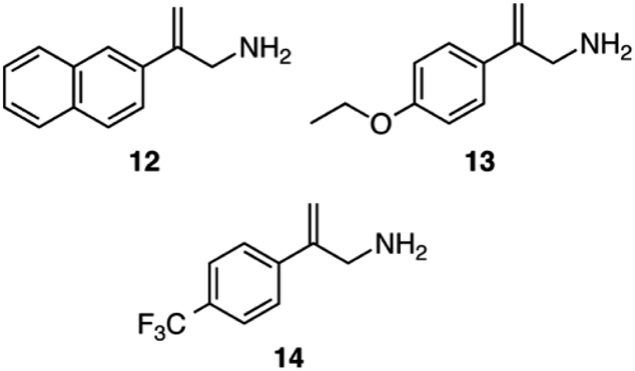
Three additional allylamines employed as substrates for asymmetric hydrogenation: 2-(naphthalene-2-yl)prop-2-en-1-amine, 12, 2-(4-ethoxyphenyl)prop-2-en-1-amine, 13, and 2-[4-(trifluoromethyl)phenyl]prop-2-en-1-amine, 14.

The asymmetric hydrogenation was also tested on three other allylamine substrates using catalysts 5, 8 and 10 (Scheme 7). For catalyst 5 the asymmetric hydrogenation was performed with CO_2_ and CyNMe_2_, whereas catalyst 8 was utilized with CO_2_ and (−)-11. For 10, both sets of conditions used for 5 and 8 were applied and found to be equally successful (Table 3).

Asymmetric hydrogenation of allylamines 6, 12, 13, and 14, utilizing catalysts 5, 8, and 10 and employing the best solvents and bases found for each^a^

**Table:** 

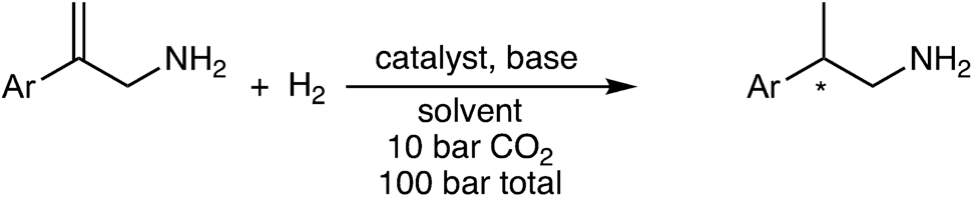		
Cat. (base + solvent)	% Yield (% ee)	
	6	12
5 (CyNMe_2_ + MeOH)	94 (73)	84 (51)
8 ((−)-11 + IPA)	88 (76)	80 (67)
10 (CyNMe_2_ + MeOH)	95 (71)	77 (62)
10 ((−)-11 + MeOH)	96 (71)	85 (66)

a Experiments were done in triplicate and at RT. Conversions for all reactions above was >95%, except as noted. The experimental error for yield% and ee% were ±10 and ±4, respectively. Reaction conditions: 160 mL stainless steel pressure vessel, 10 mg of allylamine, 10 bar CO_2_ followed by enough H_2_ pressure to bring the total pressure to 100 bar H_2(g)_, 1 eq. base, 2 mL solvent in a 1 dram vial. The reaction was stopped after 6 h. Yields are ^1^H NMR values, internal standard was 1,3,5-trimethoxybenzene. Enantiomeric excess was determined by HPLC. Catalysts 5 and 8 produced (*S*)-7, while catalyst 10 produced (*R*)*-*7.

b Conversion only 90–93%.

**Table:** 

Cat. (base + solvent)	% Yield (% ee)	
	13	14
5 (CyNMe_2_ + MeOH)	74^b^ (70)	87^b^ (28)
8 ((−)-11 + IPA)	93 (82)	41^b^ (30)
10 (CyNMe_2_ + MeOH)	88 (81)	48^b^ (27)
10 ((−)-11 + MeOH)	82 (77)	43^b^ (18)

Changing the phenyl ring of substrate 6 to a larger naphthyl ring in substrate 12 lowered the enantioselectivity by about 10% (Table 3). Adding electron-donating and electron-withdrawing groups to the *para* position of substrate 6 affected both the yield and the enantioselectivity of the allylamine. Adding an electron-donating ethoxy group (substrate 13) increased the ee to 81–82%. However, adding an electron-withdrawing trifluoromethyl group on the *para* position (substrate 14) had the opposite effect where yields and enantioselectivity both decreased; the ^1^H NMR spectrum of the reaction mixture after the reaction appeared clean and showed that the reaction was incomplete after 6 h. While the amount of product from substrate 14 might improve if the reaction time were longer, the enantioselectivity is clearly poor for this substrate.

## Conclusions

A new methodology has been developed for the asymmetric hydrogenation of allylamines. It was found that the Rh-based catASium® catalysts resulted in higher conversion and enantiomeric excess values than the Ru-binap based catalysts. Furthermore, by employing CO_2_ and an added base in the asymmetric hydrogenation of 2-phenylprop-2-en-1-amine, 6, a clean reaction was obtained, probably due to CO_2_ acting as a temporary protecting group for the amine functionality and ultimately increasing the yield of the reaction up to 94–96%. Nonetheless, the enantioselectivity of the reaction was not affected by the addition of CO_2_ and DBU. This demonstrates that the CO_2_ is not affecting the enantioselectivity-determining step and therefore the allylamine is not bound to the catalyst in the form of a carbamate ligand. The CO_2_ helps by acting as a protecting group and not by causing the allylamines to bind as carbamates to the metal centre.

Four 2-arylprop-2-en-1-amines were asymmetrically hydrogenated with the best catalysts from the above study at their optimal reaction conditions. Allylamine 2-(4-ethoxyphenyl)prop-2-en-1-amine, with an electron donating group, was hydrogenated with the greatest enantioselectivity (82% ee) and good yield (93%).

These findings demonstrate that a direct asymmetric hydrogenation of prochiral allylamines, without prior derivatization or protection, is a viable strategy for preparing chiral amines. Further optimization of the catalyst and the conditions, including the beneficial effect of CO_2_, should be able to bring the enantioselectivity to industrially useable levels.

## Experimental methods

Solvents were dried by standard distillation procedures before use. All reagents were purchased from chemical suppliers, Alfa Aesar, Sigma Aldrich, and Strem, and used as received unless otherwise specified. The four allylamines were synthesized as described in the ESI.† Glassware was dried in an oven at 110 °C before use. ^1^H NMR and ^13^C NMR spectra were recorded at 300 K on a Bruker AV-400 or AV-500 NMR spectrometer. Chemical shifts (*δ*) are expressed in ppm. Conversion and yield values were obtained through quantitative NMR spectroscopy, which was carried out using 1,3,5-trimethoxybenzene as the internal standard. Enantiomeric excess values were obtained by HPLC using Agilent Technologies 1260 Infinity with Chiralpak IA chiral column (25 cm × 0.46 cm i.d.) from Daicel. High resolution mass spectra (HRMS) ESI and EI were obtained on a Qstar XL QqTOF from Applied Biosystems/MDS Sciex.

### General racemic hydrogenation for allylamines

The allylamines were first hydrogenated with achiral catalysts in order to generate samples of the racemates for the development of instrumental methods capable of analyzing the enantiomeric mixture. The non-enantioselective hydrogenations were developed from a procedure by Hattori *et al.*^32^ The procedure below is the same for all substrates: the hydrogenation of the allylamines, Pd(5%)/CaCO_3_ was used. The hydrogenation of 2-phenylprop-2-en-1-amine, 6, is presented below and can be regarded as a general protocol for the procedure regardless of minor changes in the substrates.

To a test tube, equipped with a magnetic stir bar, 2-phenylprop-2-en-1-amine (10 mg, 75.1 mmol) and catalyst (0.5 wt% of the weight of the substrate, 2.1 mg) was added. The test tube was sealed with a rubber septum and then evacuated. THF (1 mL) was added and then hydrogen was added *via* a syringe needle from a hydrogen-filled rubber balloon (1 atm). The reaction mixture was stirred at room temperature for 5–6 h. Upon completion, the catalyst was removed by filtration through a diatomaceous earth plug, after which the solvent was removed from the product by rotatory evaporation.

### Asymmetric hydrogenation for allylamines

The following procedure was used to asymmetrically hydrogenate the allylamines. The study was completed with a variety of solvents (MeOH, IPA, and THF) with or without a non-chiral or chiral base (DBU, CyNMe_2_, iPr_2_NEt, (+)-cinchonine, (−)-cinchonidine, (+)-11, and (−)-11) with and without the presence of CO_2_, and with multiple catalysts. The hydrogenation presented below of 2-phenylprop-2-en-1-amine, 6, with (−)-4,5-bis[(2*R*,5*R*)-2,5-dimethylphospholanyl](1,2-dimethyl-1,2-dihydropyridazine-3,6-dione)(1,5-cyclooctadiene) rhodium(i) tetrafluoroborate can be regarded as a general procedure for the asymmetric hydrogenation of prochiral allylamines.

Stock solutions of the allylamine, catalyst, and the optional base were prepared in dry methanol the same day as the planned hydrogenation to ensure no decomposition of the chemicals occurred. In a 160 mL stainless steel high pressure vessel, containing up to a dozen 1 dram glass vials, each containing a magnetic stir bar, 2-phenylprop-2-en-1-amine, 6, (10 mg, 0.075 mmol), catalyst, (−)-4,5-bis[(2*R*,5*R*)-2,5-dimethylphospholanyl](1,2-dimethyl-1,2-dihydropyridazine-3,6-dione)(1,5-cyclooctadiene) rhodium(i) tetrafluoroborate, (1 mg, 0.0015 mmol) and, if desired, the optional base (*ca.* 0.075 mmol) was added under a nitrogen atmosphere. Additional dry methanol was added to each vial to obtain a total volume of 2 mL and then the vessel was sealed. The vessel was flushed 3 times with H_2_ gas or (if CO_2_ use in the experiment was planned) CO_2_ gas, and pressurized at room temperature (22 °C) to either 100 bar H_2_ gas, or if the presence of CO_2_ is desired, the vessel was pressurized to 10 bar with CO_2_ gas and then H_2_ gas was added until the total pressure was 100 bar. It is not correct to assume that the partial pressure of the H_2_ gas was equal to the difference between the total pressure and the pressure of the CO_2_ gas, because of significant CO_2_–H_2_ interactions. Once the vessel was pressurized, the reaction mixture was stirred for 6–12 h at room temperature. Once the reaction time was complete, the vessel was slowly depressurized, the solutions were filtered through diatomaceous earth, and concentrated by rotatory evaporation. Enantiomeric excess was determined by HPLC and yield was determined by ^1^H-NMR spectroscopy using an internal standard, 1,3,5-trimethoxybenzene.

#### 2-Phenylpropan-1-amine

The NMR yield was >95%. The ^1^H and ^13^C spectra matched those of the commercially available compound and those reported in the literature.^33^^1^H NMR (400 MHz, CDCl_3_): *δ* = 7.35–7.31 (m, 2H), 7.24–7.21 (m, 3H), 2.86 (d, *J* = 7.05 Hz, 2H), 2.76 (sextet, *J* = 6.92, 1H), 1.27 (d, *J* = 6.8, 3H), 1.07 (br. s, 2H) ppm; ^13^C NMR (100 MHz, CDCl_3_): *δ* = 144.85, 128.28, 127.12, 126.09, 49.35, 43.36, 19.04 ppm.

#### 2-[4-(Trifluoromethyl)phenyl]propan-1-amine

The NMR yield was >95%. ^1^H NMR (500 MHz, CDCl_3_): *δ* = 7.56 (d, *J* = 8 Hz, 2H), 7.32 (d, *J* = 8 Hz, 3H), 2.89 (dd, *J* = 12.1, 2.8 Hz, 1H), 2.85 (dd, *J* = 12.1, 2.4 Hz, 1H), 2.82 (sxt, *J* = 6.8 Hz, 1H), 1.26 (overlapping peaks, CH_3_ = d, *J* = 6.6 Hz, NH_2_ = br. s, 5H) ppm; ^13^C NMR (125 MHz, CDCl_3_): *δ* = 149.23, 128.61 (q, *J* = 32.1 Hz), 127.62, 125.36 (q, *J* = 3.8 Hz), 124.22 (q, *J* = 271.6 Hz), 49.17, 43.37, 18.95 ppm; ESI-HRMS M-calcd for C_10_H_12_F_3_N: 204.09946, found 204.09947.

#### 2-(4-Ethoxyphenyl)propan-1-amine

The NMR yield was >95%. ^1^H NMR (500 MHz, CDCl_3_): *δ* = 7.12 (d, *J* = 8.5 Hz, 2H), 6.87 (d, *J* = 8.5 Hz, 2H), 4.03 (q, *J* = 6.9 Hz, 2H), 2.84 (dd, *J* = 12.45, 6.2 Hz, 1H), 2.79 (dd, *J* = 12.45, 8.2 Hz, 1H), 2.70 (sxt, *J* = 5 × 6.8 Hz, 1H), 1.41 (t, *J* = 6.9 Hz, 3H), 1.34 (NH, br. s, 2H), 1.23 (d, *J* = 6.8 Hz, 3H) ppm; ^13^C NMR (125 MHz, CDCl_3_): *δ* = 157.47, 136.89, 128.18, 114.51, 63.40, 49.66, 12.68, 19.43, 14.89 ppm; ESI-HRMS [M + H]^+^ calcd for C_11_H_17_NO: 180.1389, found 180.1376.

#### 2-(Naphthalene-2-yl)propan-1-amine

The NMR yield was >95%. ^1^H NMR (500 MHz, CDCl_3_): *δ* = 7.83–7.80 (m, 3H), 7.66 (s, 1H), 7.49–7.43 (m, 2H), 7.38 (dd, *J* = 8.5, 1.4 Hz, 1H), 2.97–2.90 (s, sxt, *J* = 8.35 Hz, 3H) 1.36 (overlapping peaks, CH_3_ = d, NH_2_ = br. s, 5H) ppm; ^13^C NMR (125 MHz, CDCl_3_): *δ* = 142.46, 133.57, 132.38, 128.22, 127.59, 127.56, 125.98, 125.88, 125.71, 125.33, 49.40, 43.73, 19.30 ppm; ESI-HRMS M-calcd for C_13_H_15_N: 185.1209, found 185.1201.

## Conflicts of interest

There are no conflicts to declare.

## Supplementary Material

RA-012-D2RA00263A-s001
