# Supplementary material for: CO2-Assisted asymmetric hydrogenation of prochiral allylamines
Source: RSC Adv. 2022 Feb 28;12(11):6755–61. doi: 10.1039/d2ra00263a (PMC8981594; doi:10.1039/d2ra00263a)
Supplement: RA-012-D2RA00263A-s001 [file RA-012-D2RA00263A-s001.pdf]

Electronic Supplementary Material (ESI) for RSC Advances.
This journal is © The Royal Society of Chemistry 2022

Asymmetric Hydrogenation of Prochiral Allylamines

Tamara M. de Winter,[a] Jaddie Ho,[a] Christopher J. Arlidge,[a] and Philip G. Jessop[a]*

[a] Department of Chemistry, Queen's University, 90 Bader Lane, Kingston, Ontario
Fax: (+1)-613-533-6669; Phone: (+1)-613-533-3212; e-mail: Jessop@queensu.ca

Supplementary Information

Contents

1. General Remarks………………………………………………………………………….. 2
2. Preparation of α-(bromomethyl)styrene & derivatives………………...……………………….. 5
3. Preparation of 2-phenyl-3-phthalimidopropene and derivatives by Gabriel synthesis……..…….. 8
4. General procedure for the synthesis of 2-(prop-1-en-2-yl)naphthalene and 2-[2-(4-ethoxyphenyl)prop-2-en-1-yl]-1H-isoin-dole-1,3(2H)-dione…………………………………….. 10
5. Preparation of 2-phenylprop-2-en-1-amine and allylamine derivatives by deprotection..……….. 11
6. Analysis by high pressure liquid chromatography…………………………………………….. 13
7. Effect of H2 pressure, reaction time, and use of chiral bases…………………………………… 21
8. References……..………………………………………………………………………… 22

1. General Remarks

All reactions were conducted in the absence of oxygen and water under an inert atmosphere by use of standard Schlenk techniques, unless otherwise indicated. A manifold under an atmosphere of argon and a glovebag under an atmosphere of nitrogen were used for bench top manipulations of air sensitive materials. A Nexus One glovebox containing an atmosphere of nitrogen was also utilized for the preparation of high pressure reactions. All glassware and apparati were dried in an oven at 130 °C and evacuated while hot before use. Reactions carried out at room temperature (R,T) were done at 22 °C ± 2 °C. Solvents were dried by standard distillation procedures[1] before use or purchased from Drisolv® and then degassed by freeze, pump, thaw cycles. All reagents were purchased from chemical suppliers: Alfa Aesar, Sigma Aldrich, TCI America, and Acros Organics. All catalysts were purchased from Strem. Solvents, reagents and catalysts were used as received unless otherwise specified.

All asymmetric hydrogenation experiments were performed in triplicate unless indicated. ^{1}H NMR and ^{13}C NMR spectra were recorded at 300 K on a Bruker AV-400 spectrometer operating at 400.3 and 100.7 MHz and/or AV-500 NMR spectrometer operating at 499.1 and 125.5 MHz, respectively, with chemical shifts (δ) expressed in parts per million, ppm, relative to $SiMe_4$ at 0 ppm, and referenced to the residual solvent peak of the deuterated solvent. Quantitative NMR spectroscopy was carried out using 1,3,5-trimethoxybenzene as the internal standard.

Quantitative GC-FID analysis was performed on a PerkinElmer Clarus 680 gas chromatograph instrument equipped with a CP-Chirasil-DEX CB chiral column (25 m x 0.25 mm i.d., 0.25 mm film thickness) from Chrompak for the analysis of conversion and enantiomeric excess of the reactions. Low resolution mass spectrometry was done using the PerkinElmer Clarus 680 gas chromatograph paired with a Clarus 600T mass spectrometer equipped with an Elite-5MS column (25 m x 0.25 mm i.d., 0.25 mm film thickness) from PerkinElmer.

Quantitative HPLC using Agilent Technologies 1260 Infinity with Chiralpak OJ-H, AD-H and IA chiral columns (25 cm x 0.46 cm i.d.) from Daicel were also used for the analysis of enantiomeric excess.

High resolution mass spectra (HRMS) ESI and EI were obtained on a Qstar XL QqTOF from Applied Biosystems/MDS Sciex. Two of the allylamine substrates, 2-phenylprop-2-en-1-amine, **6**, and 2-(naphthalen-2-yl)prop-2-en-1-amine, **12**, were prepared as shown in scheme S1. The preparation of 2-[4-(trifluoromethyl)phenyl]prop-2-en-1-amine, **14**, is shown in scheme S2 and 2-(4-ethoxyphenyl)prop-2-en-1-amine, **13**, in scheme S3.

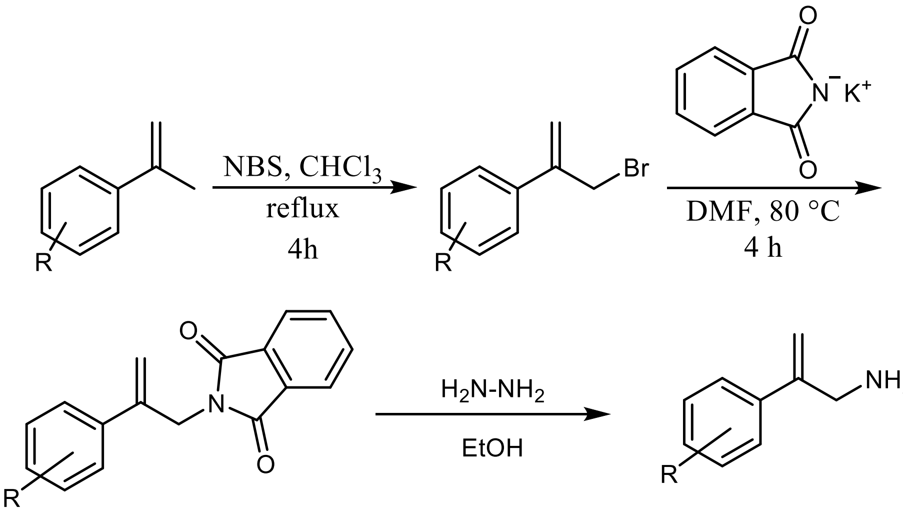

Scheme S1. Overall synthetic scheme for 2-phenylprop-2-en-1-amine, **6**, and 2-(naphthalen-2-yl)prop-2-en-1-amine, **12**.

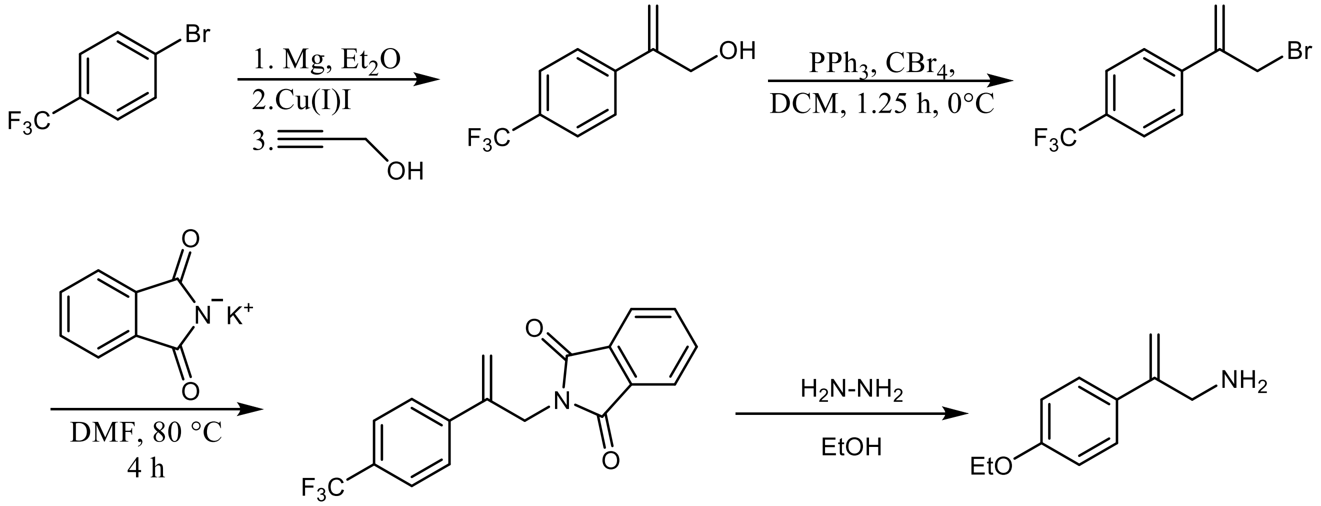

Scheme S2. Overall synthetic scheme for 2-[4-(trifluoromethyl)phenyl]prop-2-en-1-amine, **14**.

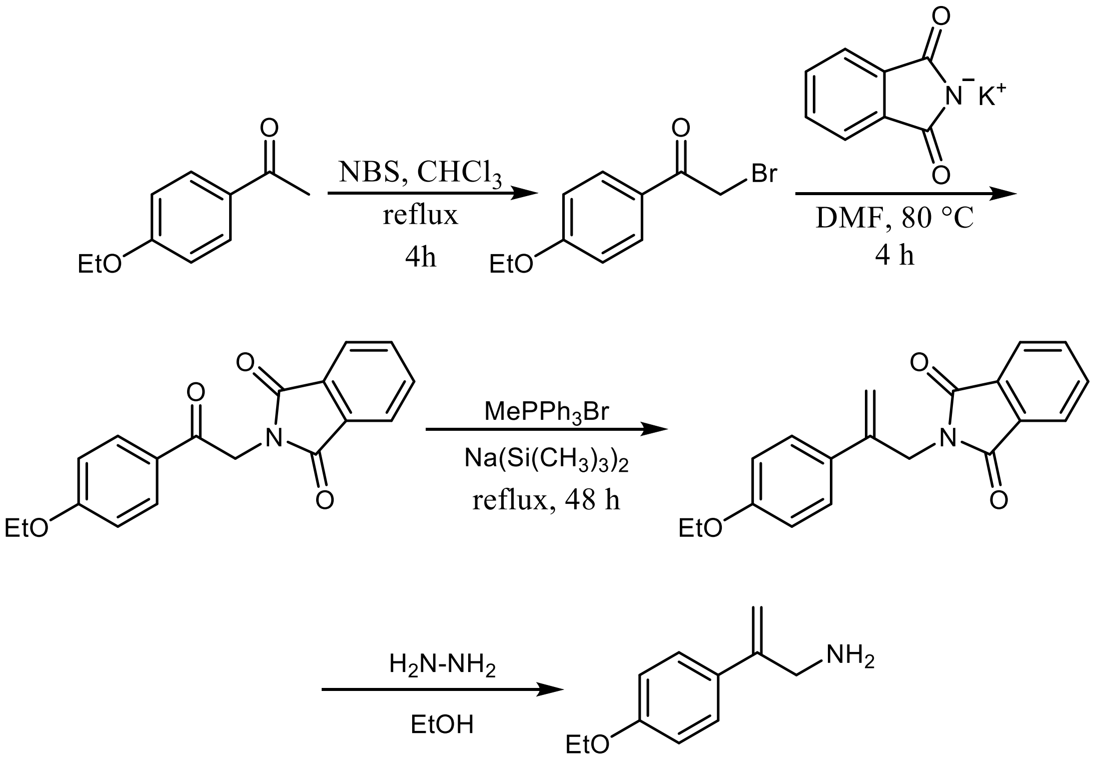

Scheme S3. Overall synthetic scheme for 2-(4-ethoxyphenyl)prop-2-en-1-amine, **13**.

2. Preparation of α-(bromomethyl)styrene & derivatives

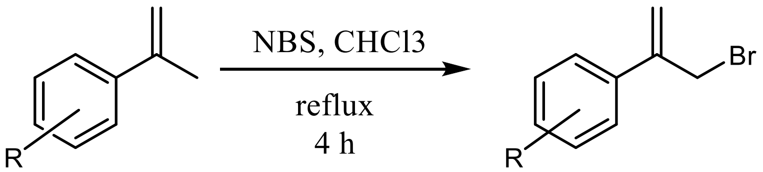

Preparation of α-(bromomethyl)styrene

The synthesis of α-(bromomethyl)styrene was adapted from the supporting information of Ohmura.[2] α-Methylstyrene (25 mL, 192 mmol) was filtered through basic alumina to remove the inhibitor, p-*tert*-butylcatechol, and rinsed three times with $CHCl_3$ (90 mL) into a round bottom flask. To the solution, N-bromosuccinimide (NBS, 39 g, 220 mmol) was added, the slurry was heated to reflux and a few drops of bromine were added. The reaction was monitored by GC-MS until completion, approximately 18 h. The reaction was cooled to room temperature and then the insoluble succinimide was removed by filtration. The filtrate was concentrated under reduced pressure and purified by column chromatography on silica gel with 15 % chloroform in hexane. The collected fractions containing product were combined and the solvent was removed by rotary evaporation to yield the pure product.

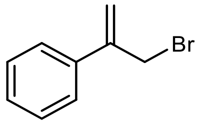

α-(Bromomethyl)styrene: The isolated yield was 65 %; clear colourless oil. The ^{1}H and ^{13}C NMR spectra matched those reported in the literature.[3] ^{1}H NMR (400 MHz, $CDCl_3$): δ = 7.52-7.50 (m, 2H), 7.42-7.33 (m, 3H), 5.58 (s, 1H), 5.51 (s, 1H), 4.40 (s, 2H) ppm; ^{13}C NMR (100 MHz, $CDCl_3$): δ = 144.24, 137.58, 128.49, 128.26, 126.08, 177.19, 34.18 ppm.

Preparation of 2-(3-bromoprop-1-en-2-yl)naphthalene

The synthesis of 2-(3-bromoprop-1-en-2-yl)naphthalene was adapted from the supporting information of Tripathi *et al.*[4] Under inert conditions, 2-(propen-2-yl)naphthalene (4.14 g, 24.6 mmol) was added to an air-free round-bottom flask, into which dry THF (100 mL) was transferred by cannula to dissolve the starting material. To the solution, N-bromosuccinimide (NBS, 4.642 g, 26.1 mmol) and p-toluenesulfonic acid (TsOH, 0.474 g, 2.5 mmol) were added. The reaction mixture was heated to a vigorous reflux (100 °C) for *ca.* 4 h. The reaction mixture was then cooled to room temperature and then petroleum ether (50-100 mL) was added. The organic was collected and washed with 3 x 100 mL of H_2O. The organic phase was then collected, dried with Na_2SO_4, filtered and concentrated under reduced pressure. Purification was done by column chromatography on silica gel using 100 % petroleum ether.

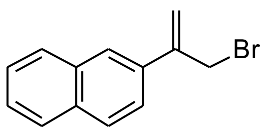

2-(3-Bromoprop-1-en-2-yl)naphthalene: The isolated yield was 56 %; clear yellow oil. ^{1}H NMR (400 MHz, $CDCl_3$): δ = 7.96 (s, 1H), 7.89-7.83 (m, 3H), 7.65-7.63 (m, 1H), 7.53-7.48 (m, 2H), 5.72 (s, 1H), 5.61 (s, 1H), 4.51 (s, 1H) ppm; ^{13}C NMR (100 MHz, $CDCl_3$): δ = 144.16, 134.79, 133.30, 133.17, 128.41, 128.22, 127.63, 126.41, 126.36, 125.29, 124.07, 117.66, 34.23 ppm; EI-HRMS $[M^-]$ calcd for $C_{13}H_{11}Br$ (Isotope 79): 246.0049, found 246.0044.

Preparation of 2-bromo-1-(4-ethoxyphenyl)ethanone

The synthesis of 2-bromo-1-(4-ethoxyphenyl)ethanone was adapted from the methods of Tripathi *et al.* [4] and Mohan Reddy *et al.*[5] To a round bottom flask equipped with a magnetic stir bar, 4'-ethoxyacetophenone (5.28 g, 32.1 mmol), *p*-toluenesulfonic acid (0.1 mol equivalents, 0.614 g, 3.21 mmol) and *ca.* 1/6th of the needed n-bromosuccinimide, NBS, (1.05 mol equivalents, 6.01 g, 33.7 mmol) was added. Methanol, 100 mL, was then added and the reaction was refluxed at 65 °C for 3 h. Five further additions of NBS, for a total of 6 additions, were added at 25-30 min intervals. The methanol was removed by rotatory evaporation. Then to the product aqueous sodium thiosulfate was added, *ca.* 100 mL, and the product was extracted using CH_2Cl_2, 3 x 50 mL. The organic layers were collected and washed with 3 x 100 mL of H_2O. The organic phase was then collected, dried with Na_2SO_4, filtered and concentrated under reduced pressure. Purification was done by column chromatography on silica gel using 100 % CH_2Cl_2. The collected fractions containing product were combined and concentrated by rotary evaporation to yield the product.

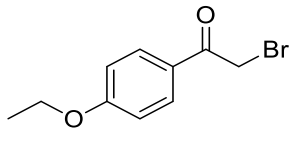

2-Bromo-1-(4-ethoxyphenyl)ethanone: The isolated yield was 88 %; white solid. ^{1}H NMR (400 MHz, $CDCl_3$): δ = 7.97 (d, 2H), 6.95 (d, 2H), 4.41 (s, 2H), 4.13 (q, 2H), 1.46 (t, 3H) ppm; ^{13}C NMR (100 MHz, $CDCl_3$): δ = 189.89, 163.56, 131.32, 126.69, 114.45, 63.87, 30.69, 14.60 ppm; EI-HRMS [M-] calcd for $C_{10}H_{11}BrO_2$ (Isotope 79): 241.9947, found 241.9949.

Preparation of 2-[4-(trifluoromethyl)phenyl]prop-2-en-1-ol

The synthesis of 2-[4-(trifluoromethyl)phenyl]prop-2-en-1-ol was adapted from the methods of Garzan *et al.*[6] and Duan *et al.*[7] To a round bottom flask equipped with a magnetic stir bar, $Mg_{(s)}$ (0.868 g, 35.7 mmol) was added and then the system was flame-dried and placed under inert conditions. Drisolv® diethyl ether, 64 mL, was added and then the system was cooled to 0 °C using an ice bath. The aryl bromide, 4-bromobenzotrifluoride (5.0 mL, 8.035 g, 35.7 mmol), was added slowly in a drop-wise fashion; once the addition was completed the reaction mixture was refluxed for 2.25 h, until all the magnesium chunks disappeared. The reaction was then cooled to room temperature. Copper (I) iodide (0.15 equivalents, 0.408 g, 2.14 mmol) was added and the mixture stirred for 0.5 -0.75 h, until the solid copper (I) iodide was gone. Propargyl alcohol (0.4 equivalents, 0.83 mL, 0.801 g, 14.3 mmol) in 20 mL Drisolv® diethyl ether was then added slowly in a drop-wise manner to the solution. Once the addition was done, the reaction was heated to reflux for 24 h. After cooling to room temperature, the solution was quenched using saturated $NH_4Cl_{(aq)}$ solution which was slowly added until the solution stopped reacting. The organic phase was separated from the aqueous phase, which was further extracted using diethyl ether (4-6 x 50-75 mL) until the aqueous phase went from brown to blue. The collected organic fractions were combined, washed with brine, and dried using anhydrous $MgSO_4$. The solvent was removed by rotary evaporation and the product purified by column chromatography using 10 % ethyl acetate in hexanes and slowly increasing the eluent to 15 % ethyl acetate in hexanes.

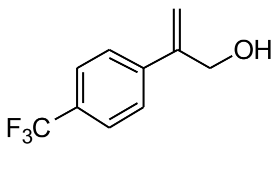

2-[4-(Trifluoromethyl)phenyl]prop-2-en-1-ol: The isolated yield was 98 %; reddish orange oil . The ^1H and ^{13}C NMR spectra matched literature.[6] ^1H NMR (400 MHz, $CDCl_3$): δ = 7.61 (d, 2H), 7.55 (d, 2H), 5.55 (s, 1H), 5.56 (d, 1H), 4.54 (s, 2H), 2.07 ppm (broad s, 1H); ^{13}C NMR (100 MHz, $CDCl_3$): δ = 146.11, 142.08, 129.84 (q, J = 32.65 Hz), 126.37, 125.37 (q, J = 3.67 Hz), 124.10 (q, J = 271.8), 114.72, 64.69 ppm.

Preparation of 1-(3-bromoprop-1-en-2-yl)-4-(trifluoromethyl)benzene

The synthesis of 1-(3-bromoprop-1-en-2-yl)-4-(trifluoromethyl)benzene was adapted from the methods of Garzan *et al.*[6] and Baumgartner *et al.*[8] To a flame dried round bottom flask equipped with a magnetic stir bar, a solution of 2-[4-(Trifluoromethyl)phenyl]prop-2-en-1-ol (8.05 g, 39.8 mmol) in Drisolv® DCM, 75 mL, was prepared and cooled to 0 °C using an ice bath. Triphenylphosphine (1.2 equivalents, 12.5 g, 47.8 mmol) was first added and then CBr_4 (1.1 equivalents, 14.5 g, 43.8 mmol) was added slowly to the reaction mixture. The mixture was stirred at 0 °C for 1.25 h; the reaction was checked by GC-MS for completion. The solvent was removed by reduced pressure and the crude product was purified by column chromatography using 10 % ethyl acetate in hexane as the eluent.

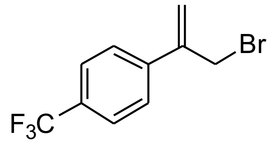

1-(3-Bromoprop-1-en-2-yl)-4-(trifluoromethyl)benzene: The isolated yield was 98 %; clear yellow liquid . The ^{1}H and ^{13}C NMR spectra matched literature.[6] ^{1}H NMR (400 MHz, $CDCl_3$): δ = 7.65 (d, 2H), 7.61 (d, 2H), 5.63 (s, 1H), 5.61 (s, 1H), 4.39 (s, 2H) ppm; ^{13}C NMR (100 MHz, $CDCl_3$): δ = 143.21, 141.15, 130.14 (q, J = 32.65 Hz), 126.45, 125.43 (q, J = 3.67 Hz), 124.06 (q, J = 271.8 Hz), 118.93, 33.41 ppm.

3. Preparation of 2-phenyl-3-phthalimidopropene and derivatives by Gabriel synthesis

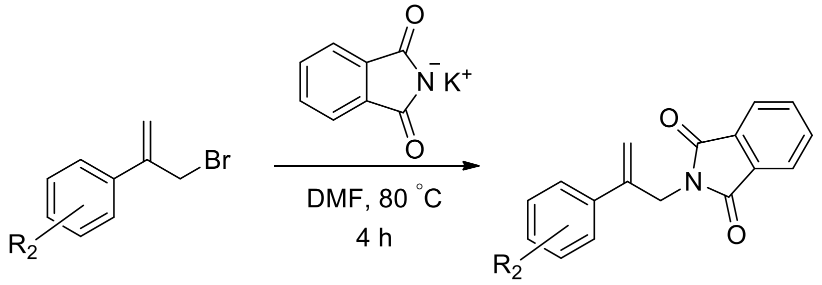

Preparation of 2-phenyl-3-phthalimidopropene and derivatives by Gabriel synthesis

The synthesis of 2-phenyl-3-phthalimidopropene and derivatives followed the synthesis by Dumas[9] with a slight variation. To a solution of α-(bromomethyl)styrene (24.61 g, 124.9 mmol) dissolved in 100 mL of DMF, potassium phthalimide (1.11 equivalents, 25.71 g, 138.8 mmol) was added. The reaction mixture was heated to 82-83 °C and monitored by GC-MS until completion, *ca.* 1-19 h. Once the reaction had come to completion, the hot solution was poured onto ice and a light yellow precipitate formed. The precipitate was collected by vacuum filtration and recrystallized using hot ethanol. White crystalline shards were collected by vacuum filtration and left to dry under vacuum for an hour.

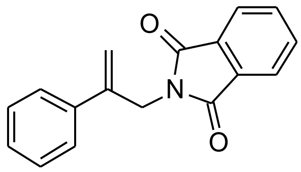

2-Phenyl-3-phthalimidopropene: Reaction time: 19 hours. The isolated yield was 87 %; white crystals. The ^{1}H and ^{13}C NMR spectra matched those reported in the literature.[10,11] ^{1}H NMR (400 MHz, $CDCl_3$): δ = 7.86-7.85 (m, 2H), 7.73-7.71 (m, 2H), 7.51 (d, 2H), 7.35 (t, 2H), 7.31-7.29 (m, 1H), 5.45 (s, 1H), 5.17 (s, 1H), 4.72(s 2H) ppm; ^{13}C NMR (100 MHz, $CDCl_3$): δ = 167.94, 142.41, 138.51, 133.99, 132.01, 128.39, 128.03, 126.38, 123.55, 113.87, 41.44 ppm.

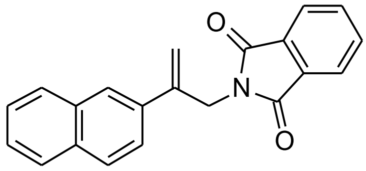

2-[2-(Naphthalen-2-yl)prop-2-en-1-yl]-1*H*-isoind-ole-1,3(2*H*)-dione: Reaction time: 3 hours. The isolated yield was 76 %; white fine crystalline shards. ^{1}H NMR (500 MHz, $CDCl_3$): δ = 7.97 (s, 1H), 7.87-7.80 (m, 5H), 7.71-7.69 (m, 2H), 7.66 (dd, 1H), 7.50-7.45 (m, 2H), 5.61 (s, 1H), 5.30 (s, 1H), 4.85 (s, 2H) ppm; ^{13}C NMR (125 MHz, $CDCl_3$): δ = 167.98, 142.24, 135.65, 133.98, 133.20, 132.99, 131.98, 128.30, 127.98, 127.50, 126.22, 126.11, 125.26, 124.56, 123.35, 144.59, 41.50 ppm; EI-HRMS [M^-] calcd for $C_{21}H_{15}NO_2$: 313.1108, found 313.1109.

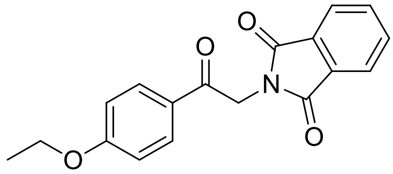

2-[2-(4-Ethoxyphenyl)-2-oxoethyl]-1*H*-isoind-ole-1,3(2*H*)-dione: Reaction time: 1 hours. The isolated yield was 70 %; white crystalline shards. ^{1}H NMR (500 MHz, $CDCl_3$): δ = 7.98 (d, 2H), 7.90-7.88 (m, 2H), 7.75-7.74 (m, 2H), 6.96 (d, 2H), 5.09 (s, 2H), 4.12 (q, 2H), 1.45 (t, 3H) ppm; ^{13}C NMR (125 MHz, $CDCl_3$): δ = 189.26, 167.93, 163.57, 134.01, 132.25, 130.41, 127.22, 123.45, 114.45, 63.83, 43.82, 14.58 ppm; EI-HRMS [M-] calcd for $C_{18}H_{15}NO_4$: 309.1007, found 309.1008.

2-{2-[4-(Trifluoromethyl)phenyl]prop-2-en-1-yl}-1*H*-isoindole-1,3(2*H*)-dione: Reaction time: 0.5 hour. The isolated yield was 70 %; recrystallized using hot 95 % ethanol, white crystals were obtained. The ^{1}H and ^{13}C NMR matched literature.[6] ^{1}H NMR (400 MHz, $CDCl_3$): δ = 7.86 (m, 2H), 7.73 (m, 2H), 7.61 (m, 4H), 5.53 (s, 1H), 5.33 (s, 1H), 4.72 (s, 2H) ppm; ^{13}C NMR (100 MHz, $CDCl_3$): δ = 167.80, 141.97, 141.50, 134.05, 131.83, 129.94 (q, J = 32.6 Hz), 126.71, 125.32 (q, J = 3.67 Hz), 124.01 (q, J = 271.8 Hz), 123.35, 116.31, 41.17 ppm; EI-HRMS [M-] calcd for $C_{18}H_{12}F_3NO_2$: 331.0826, found 331.0828.

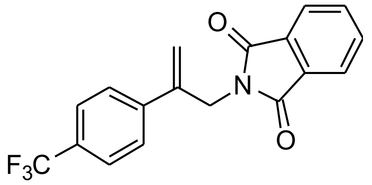

4. General procedure for the synthesis of 2-(prop-1-en-2-yl)naphthalene and 2-[2-(4-ethoxyphenyl)prop-2-en-1-yl]-1H-isoin-dole-1,3(2H)-dione

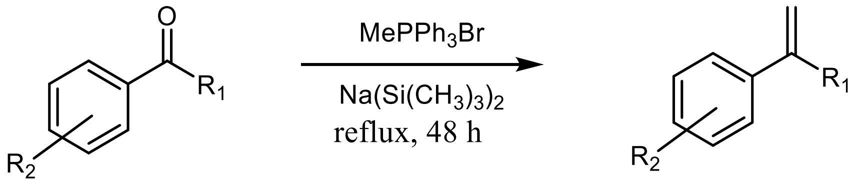

Methyltriphenylphosphonium bromide (5.6 mmol) was suspended in toluene, where it was subsequently cooled to 0 °C in an ice bath. Sodium bis(trimethylsilyl)amide (5.4 mmol) in a 1.0 M solution in THF was added drop-wise to the suspension and rapidly stirred for 1 h. The suspension was then cooled from 0 °C to ca. -78 °C using a dry ice/acetone bath, where 2-acetophenone (5.2 mmol) was added to the solution. The reaction was then refluxed for 16-48 h until the reaction came to completion, which was monitored by GC-MS. Upon cooling, saturated ammonium chloride (80-100 mL) was added to the reaction flask and the resulting slurry was diluted with distilled water (100 mL). The product was extracted with ethyl acetate (3 x 100 mL), washed with brine, and dried with magnesium sulfate. The drying agent was removed by filtration, after which the product was concentrated under reduced pressure by rotary evaporation and purified by column chromatography on silica gel with 30 % ethyl acetate in hexane.

2-(Prop-1-en-2-yl)naphthalene: The isolated yield was 77 % of a white crystalline solid. The ^{1}H and

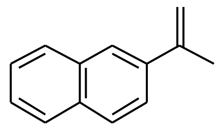

^{13}C spectra matched those reported in the literature.[12] ^{1}H NMR (400 MHz, $CDCl_3$): δ = 7.87-7.80 (m, 4H), 7.71-7.68 (m, 1H), 7.51-7.44 (m, 2H), 5.55 (s, 1H), 5.22 (t, 1H), 2.29 (s, 3H) ppm; ^{13}C NMR (100 MHz, $CDCl_3$): δ = 142.98, 138.32, 133.36, 132.78, 128.21, 127.66, 127.48, 126.08, 125.79, 124.24, 123.87, 112.99, 21.86 ppm.

2-[2-(4-Ethoxyphenyl)prop-2-en-1-yl]-1H-isoin-dole-1,3(2H)-dione: The isolated yield was 74 % of white crystalline needle-like crystals. ^{1}H NMR (500 MHz, $CDCl_3$): δ = 7.85-7.83 (m, 2H), 7.71-7.69

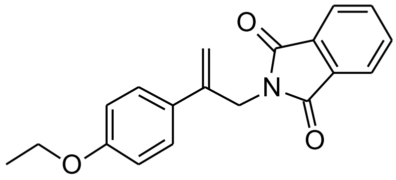

(m, 2H), 7.43 (d, 2H), 6.86 (d, 2H), 5.37 (s, 1H), 5.08 (s, 1H), 4.68 (s, 2H), 4.03 (q, 2H), 1.40 (t, 3H) ppm; ^{13}C NMR (125 MHz, $CDCl_3$): δ = 167.93, 158.83, 141.70, 133.92, 131.99, 130.67, 127.45, 123.28, 114.23, 112.27, 63.35, 41.44, 14.75 ppm. EI-HRMS [M-] calcd for $C_{19}H_{17}NO_3$: 307.1213, found 307.1211.

5. Preparation of 2-phenylprop-2-en-1-amine and allylamine derivatives by deprotection

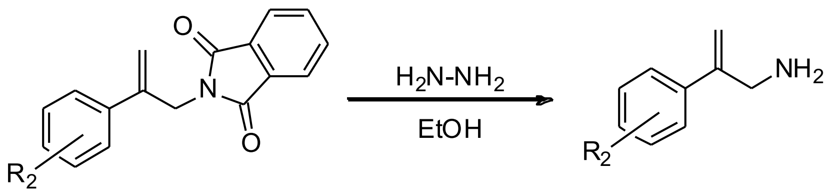

The synthesis was modified from the preparation of Dumas.[9] To a slurry of 2-phenyl-3-pthalimidopropene (24.71 g, 93.85 mmol) in 200 mL of ethanol was slowly added hydrazine hydrate (2 mol equivalence, 50-60 % solution, 14 mL) to the mixture at room temperature. The reaction mixture was placed in a hot oil bath heated to 80 - 87 °C. The white solid in the slurry dissolved and the reaction mixture became a clear yellow solution. The resulting solution was refluxed for *ca.* 30 minutes and then cooled to room temperature. As the reaction cooled, a white precipitate formed. Once cooled, 350 mL of 1 N HCl was added to the slurry and refluxed until the solution became clear or for *ca.* 5 minutes if the precipitate never re-dissolved. The reaction mixture was then cooled, and 2,3-dihydro-1,4-phthalazinedione precipitated and was removed by filtration and washed with a copious amount of H_2O. The filtrate was collected and the amine salt was obtained by rotary evaporation. The salt was purified by recrystallization in 2-propanol. The crystals were collected by vacuum filtration, re-dissolved in H_2O, and base treated with a concentrated solution of NaOH. Once the aqueous solution obtained a high pH, the 2-phenylprop-2-en-1-amine was extracted with *ca.* 200 mL $CHCl_3$, the combined organic fractions were dried with $MgSO_4$ and concentrated by rotary evaporation.

The reaction time varied depending on the size of the reaction and the choice of allylamine.

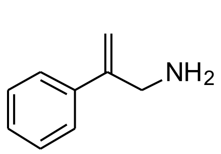

2-Phenylprop-2-en-1-amine, 6: The isolated yield was 86 %; clear and colorless liquid. The ^{1}H and ^{13}C NMR spectra matched those reported in the literature.[6] ^{1}H NMR (400 MHz, $CDCl_3$): δ = 7.43 (d, 2H), 7.36, (t, 2H), 7.30 (m, 1H), 5.36 (s, 1H), 5.24 (s, 1H), 3.73 (s, 2H), 1.28 (s, 2H, NH) ppm; ^{13}C NMR (100 MHz, $CDCl_3$): δ = 149.73, 139.71, 128.39, 127.59, 126.04, 111.12, 46.06 ppm.

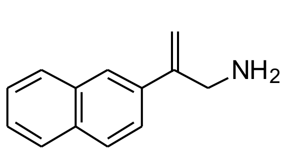

2-(Naphthalen-2-yl)prop-2-en-1-amine, 12: The isolated yield was 76 %; whitish yellow solid. ^{1}H NMR (400 MHz, $CDCl_3$): δ = 7.86-7.82 (m, 4H), 7.60 (dd, 1H), 7.52-7.46 (m, 2H), 5.52 (s, 1H), 5.35 (s, 1H), 3.85 (s, 2H), 1.35 (br. s, 2H) ppm; ^{13}C NMR (100 MHz, $CDCl_3$): δ = 149.58, 136.95, 133.36, 132.89, 128.12, 128.05, 127.52, 126.19, 125.93, 124.65, 124.56, 111.80, 46.18 ppm; EI-HRMS [M-] calcd for $C_{13}H_{13}N$: 183.1053, found 183.1051.

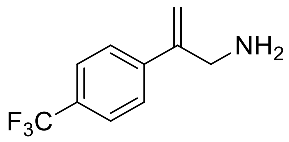

2-[4-(Trifluoromethyl)phenyl]prop-2-en-1-amine, 14: The isolated yield was 35 %; clear colorless oil. ^{1}H NMR (500 MHz, $CDCl_3$): δ =7.61 (d, 2H), 7.53 (d, 2H), 5.44 (s, 1H), 5.36 (s, 1H), 3.75 (s, 2H), 1.31 (s, 2H, NH) ppm; ^{13}C NMR (100 MHz, $CDCl_3$): δ = 148.5, 143.4 (q, J = 1.2 Hz), 129.53 (q, J = 32.6 Hz), 126.34, 125.27 (q, J = 3.67 Hz), 124.08 (q, J = 271.8 Hz), 113.13, 45.83 ppm; the carbon NMR peaks were determined using Heteronuclear Single Quantum Coherence, HSQC, spectroscopy and Heteronuclear Multiple-bond Correlation, HMBC, spectroscopy. EI-HRMS [M+] calcd for $C_{10}H_{10}F_3N$: 201.0760, found 201.0761.

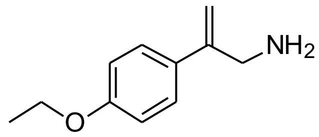

2-(4-Ethoxyphenyl)prop-2-en-1-amine, 13: The isolated yield was 25 %; clear yellow oil. ^{1}H NMR (400 MHz, $CDCl_3$): δ = 7.37 (d, 2H), 6.90 (d, 2H), 5.30 (s, 1H), 5.16 (s, 1H), 4.07 (q, 2H), 3.71 (s, 2H), 1.44 (t, 3H), 1.49 (br. s, 2H, NH) ppm; ^{13}C NMR (100 MHz, $CDCl_3$): δ = 158.58, 148.94, 131.82, 127.09, 114.35, 109.60, 63.38, 46.05, 14.77 ppm; EI-HRMS [M-] calcd for $C_{11}H_{15}NO$: 177.1159, found 177.1159.

6. Analysis by high pressure liquid chromatography

The hydrogenated samples were filtered through diatomaceous earth and a 0.2 µm HPLC filter before being analyzed on the Agilent technologies 1260 infinity HPLC equipped with either a Chiralpak IA chiral columns (25 cm x 0.46 cm i.d.) from Daicel. The following methods, listed below, were used for the analysis of enantiomeric excess.

Method 1 for HPLC analysis of 2-phenylpropan-1-amine, 6.

The analysis of 2-phenylpropan-1-amine was done using a Chiralpak IA chiral column (25 cm x 0.46 cm i.d.) from Daicel. The mobile phase was 97:3 n-hexane:*iso*-propanol with an additive of 0.01 % ethylenediamine. The flow was 0.7 mL/min.

Method 2 for HPLC analysis of 2-(naphthalene-2-yl)propan-1-amine, 2-(4-ethoxyphenyl)propan-1-amine, and 2-[4-trifluoromethyl)phenyl]propan-1-amine

The analysis of 2-(naphthalene-2-yl)propan-1-amine, 2-(4-ethoxyphenyl)propan-1-amine, and 2-[4-(trifluoromethyl)phenyl]propan-1-amine was done using a Chiralpak IA chiral column (25 cm x 0.46 cm i.d.) from Daicel. The mobile phase was 98.5-98.6 % n-hexane: 1.5-1.4 % *iso*-propanol with an additive of 0.01 % ethylenediamine. The flow was 0.6 mL/min.

NMR Spectral Data

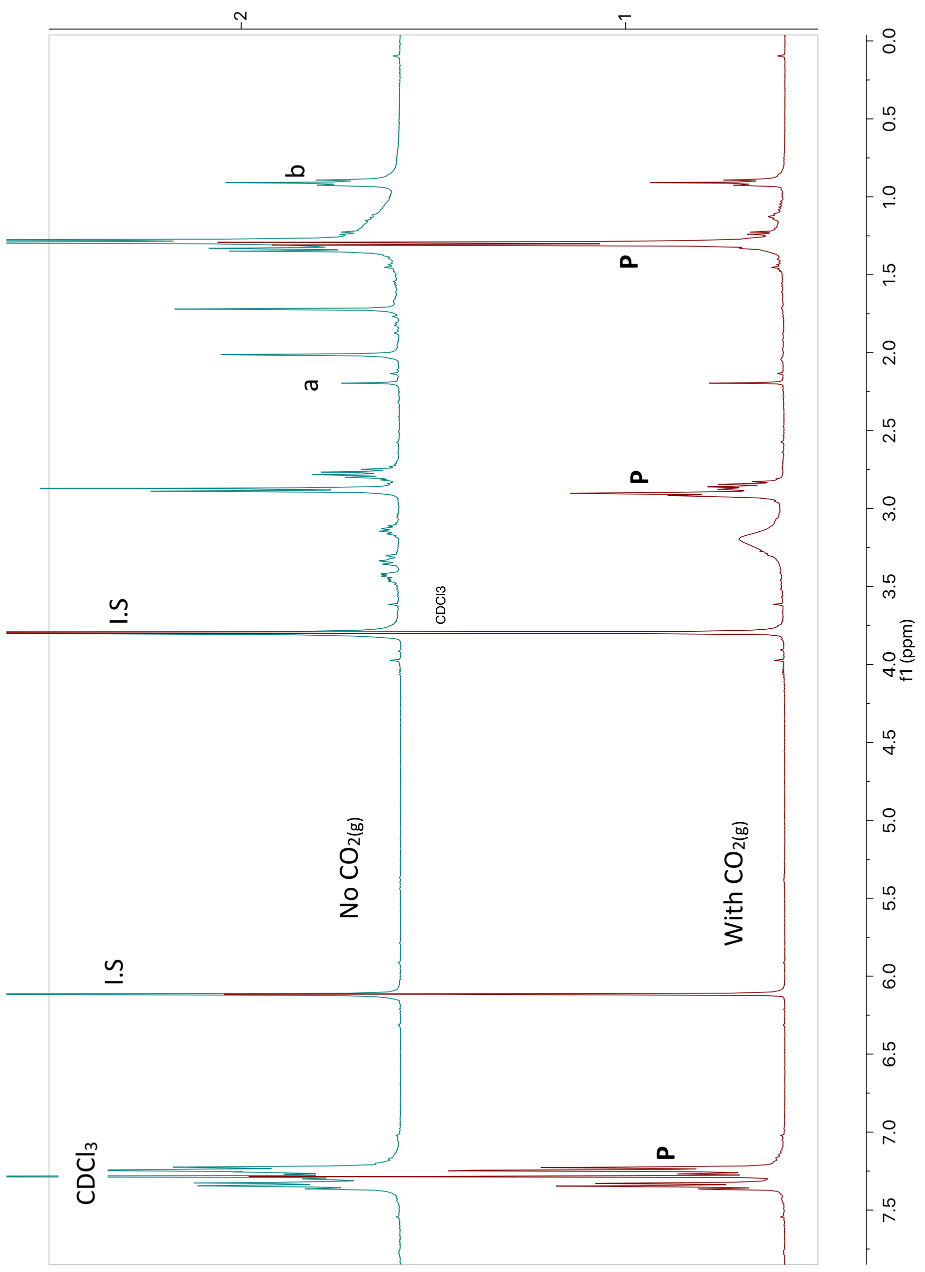

Fig S1. ^{1}H NMR spectra of 2-phenylpropan-1-amine (**P**) after the asymmetric hydrogenation with **5** without base and 1,3,5-trimethoxybenzene as the NMR internal standard (I.S.) (acetone = a, hexane = b). The green spectrum represents the hydrogenation without $CO_{2(g)}$ and the red spectrum represents the hydrogenation with $CO_{2(g)}$.

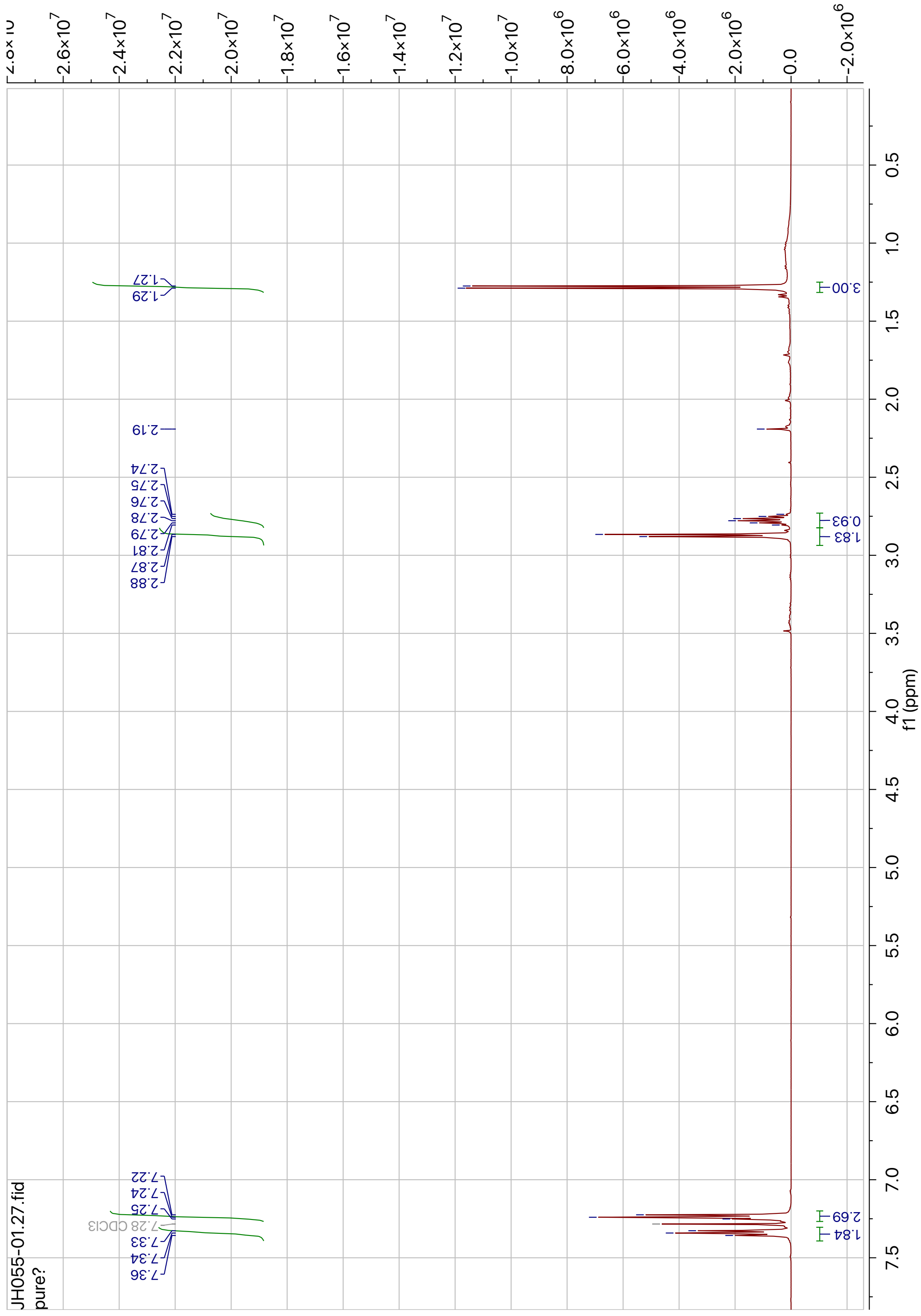

Fig S2. ^{1}H NMR for isolated yield of **6** after purification. Peak at 2.19 ppm is residual acetone on the NMR tube from cleaning.

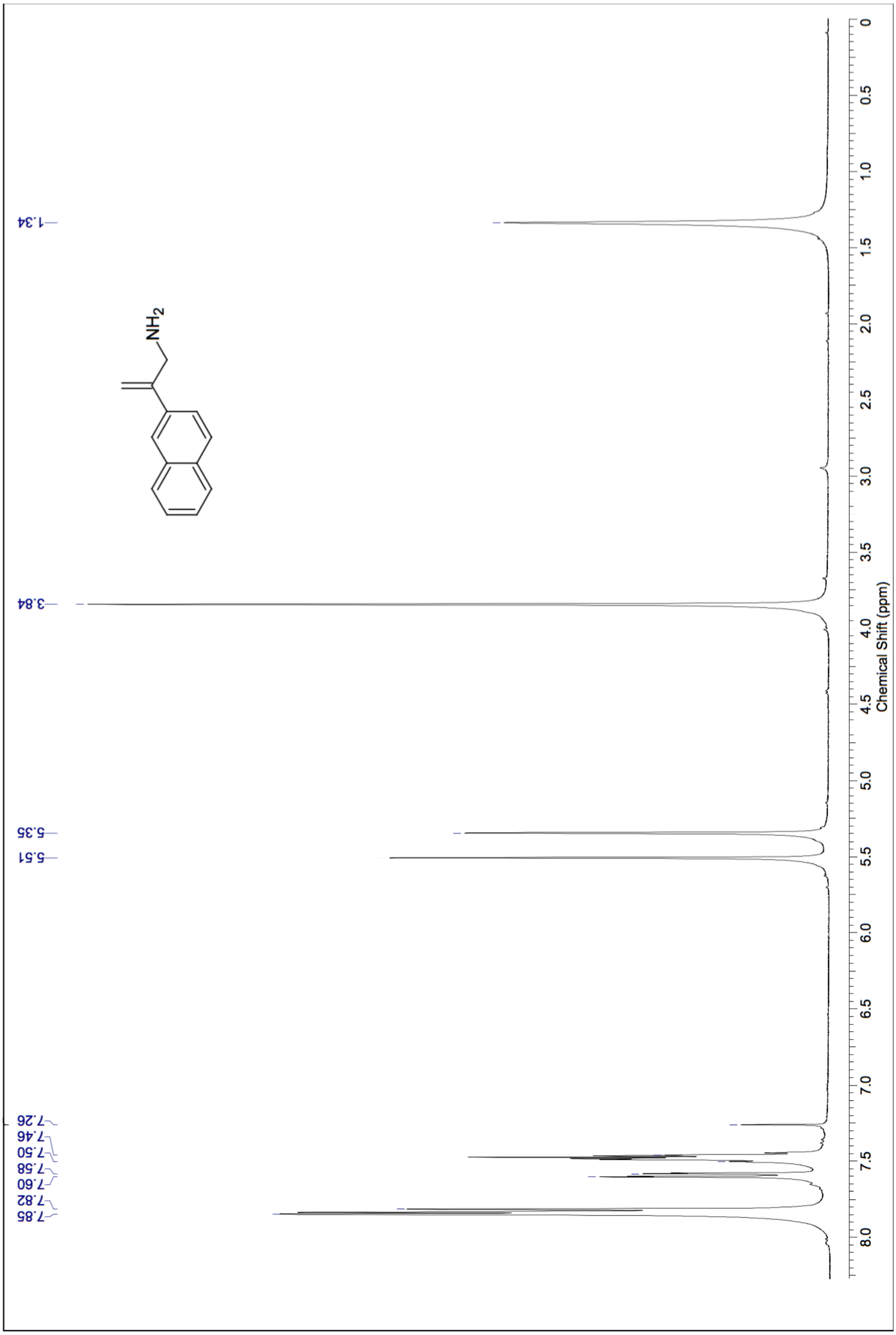

Fig S3. ^{1}H spectra of isolated 2-(Naphthalen-2-yl)prop-2-en-1-amine

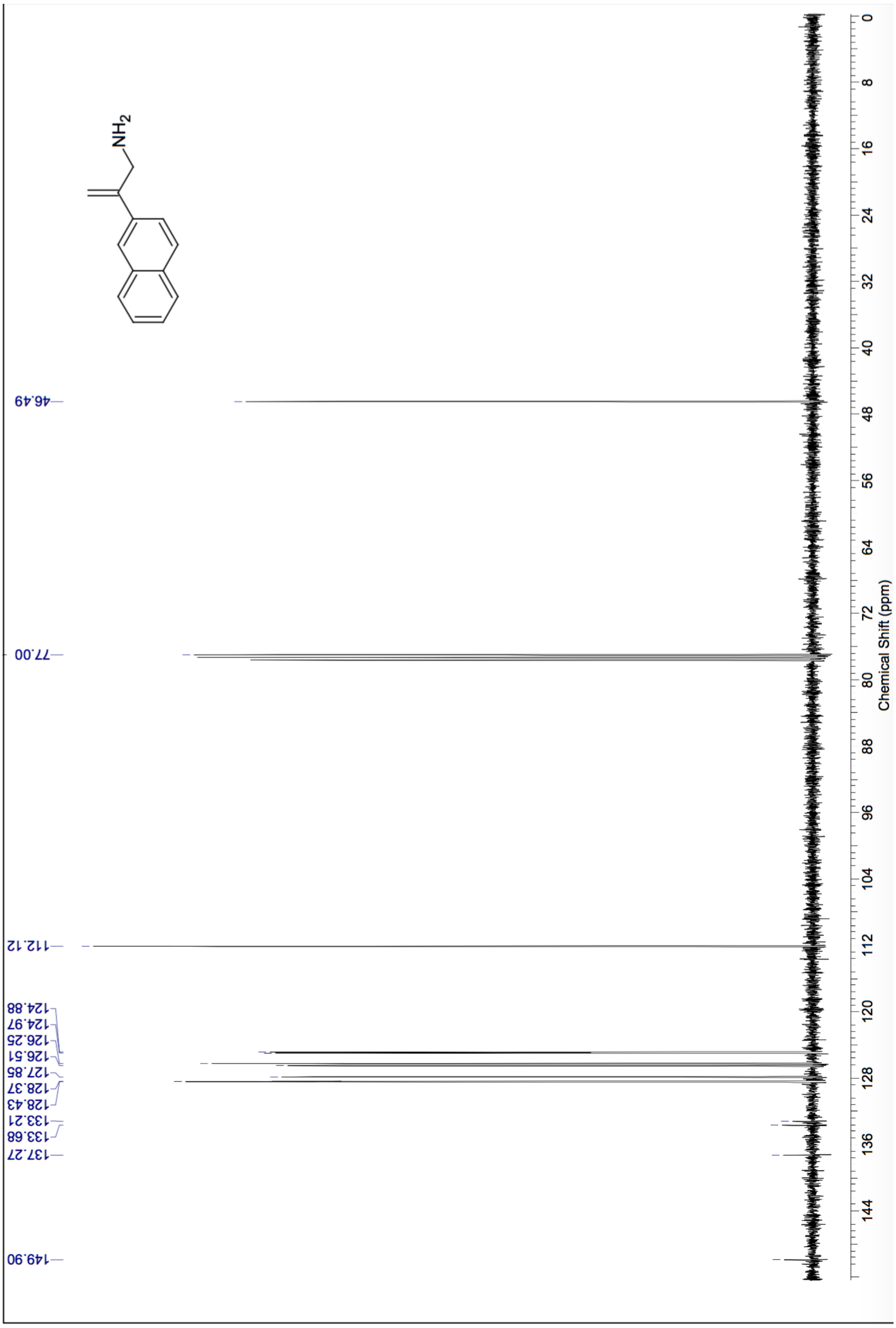

Fig S4. ^{13}C spectra of isolated 2-(Naphthalen-2-yl)prop-2-en-1-amine

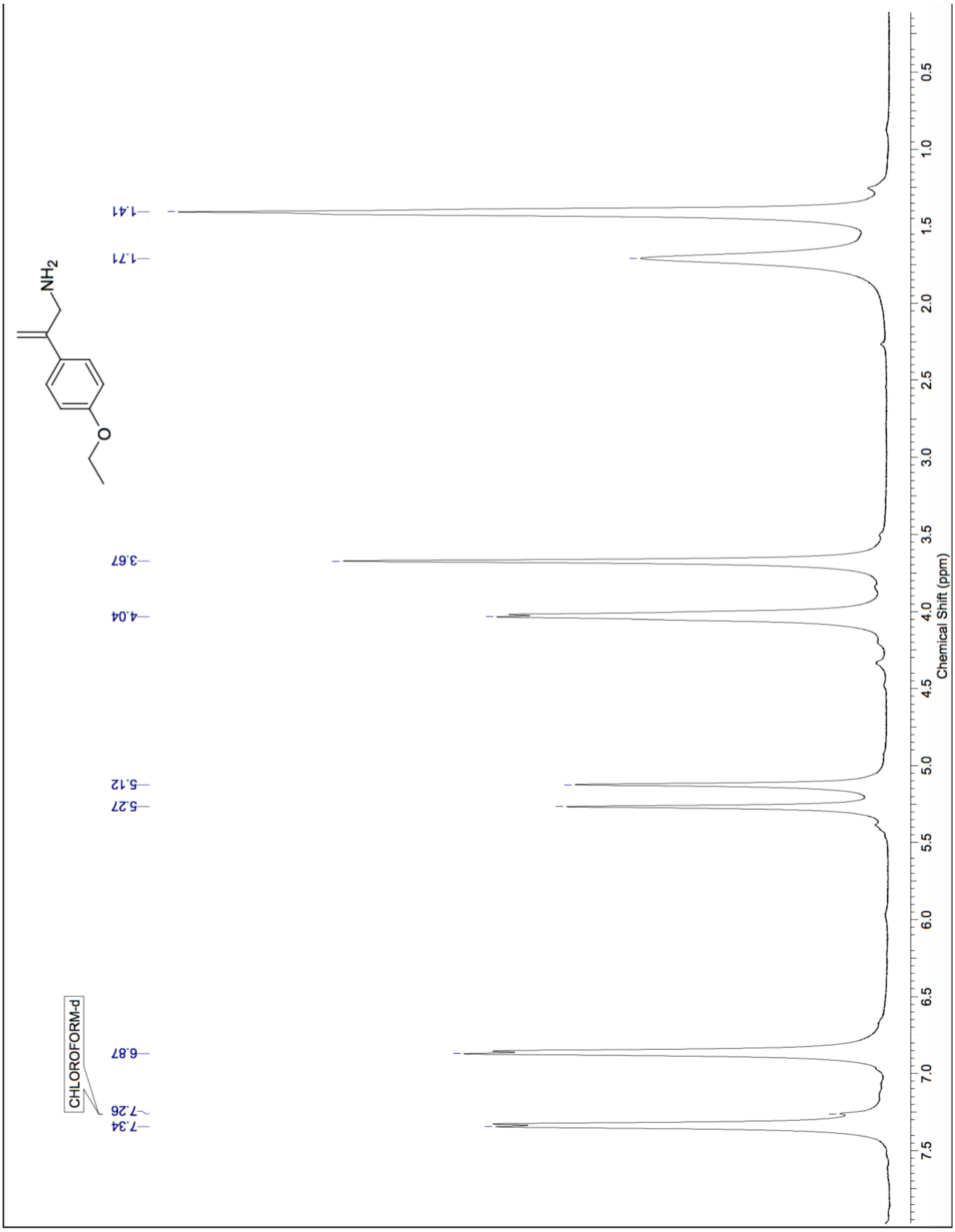

Fig S5. ^{1}H spectra of isolated 2-(4-Ethoxyphenyl)prop-2-en-1-amine

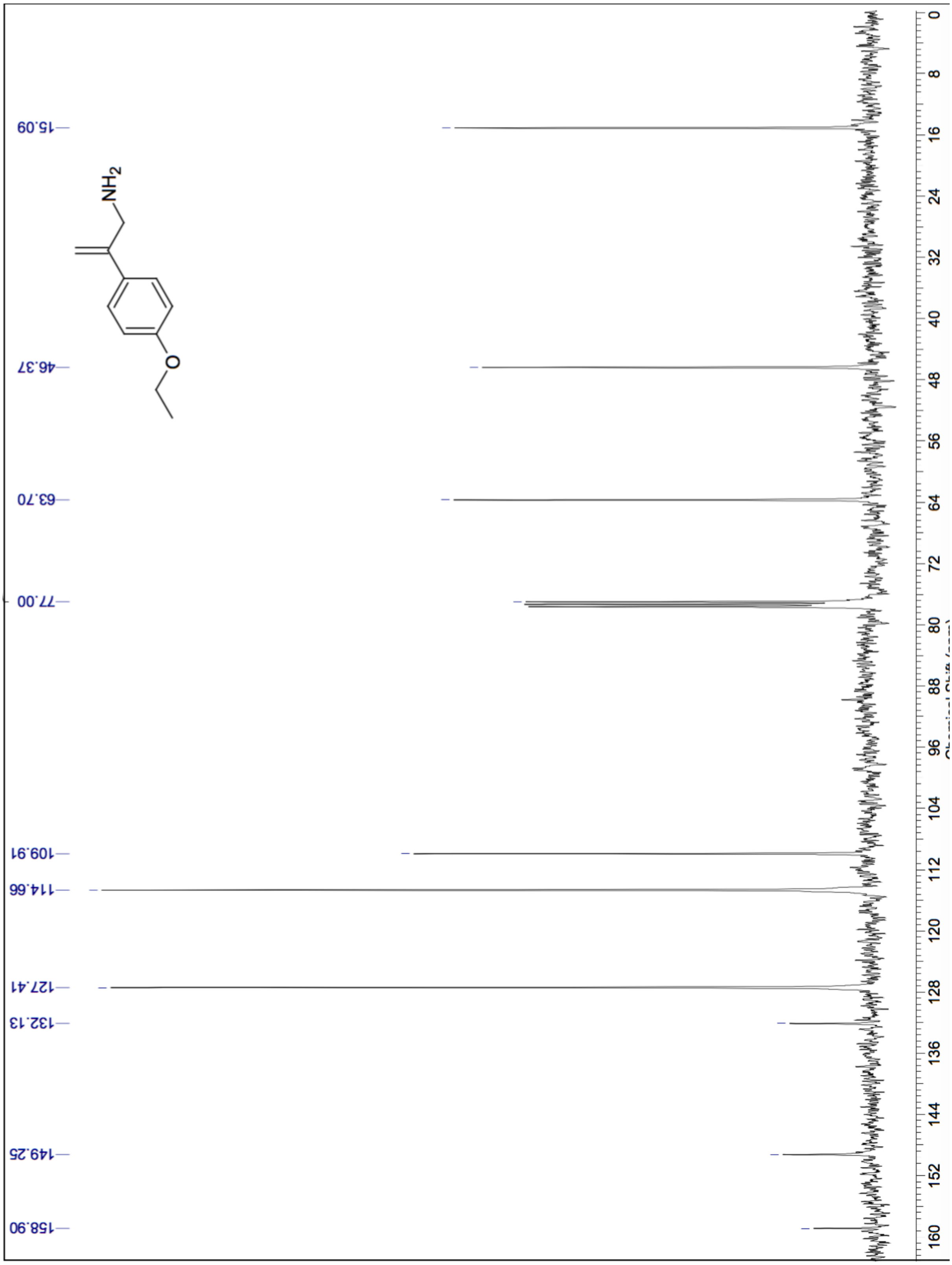

Fig S6. ^{13}C spectra of isolated 2-(4-Ethoxyphenyl)prop-2-en-1-amine

7. Effect of H_2 pressure, reaction time, and use of chiral bases

Table S1. Asymmetric hydrogenation results for 2-phenylprop-2-en-1-amine, **6**, comparing the four rhodium(I) catalysts, **5**, **8**, **9**, and **10** at high pressure and low pressure of $H_{2(g)}$, and $CO_{2(g)}$.[a)]

		20 bar total	100 bar total
Additive	Cat.	% yield (% ee)	% yield (% ee)
none [b)]	**5**	79 (63)	57 (68)
	**8**	79 (70)	66 (61)
	**9**	74 (39)	47 (24)
	**10**	-	54 (71)
DBU [c)]	**5**	56 (33)	50 (26)
	**8**	79 (54)	71 (69)
	**9**	67 (2)	48 (20)
	**10**	-	52 (36)
CO_2 [d)]	**5**	88 (61)	84 (75)
	**8**	79 (72)	54 (65)
	**9**	72 (42)	57 (23)
	**10**	-	90 (70)
CO_2 + DBU [e)]	**5**	76 (66)	94 (73)
	**8**	71 (69)	72 (69)
	**9**	58 (43)	49 (22)
	**10**	-	69 (76)

[a)] Experiments were done in triplicate and at RT in a 160 mL stainless steel vessel containing 10 mg **6** and 2 mL methanol in a 1 dram vial under 100 bar total pressure. Reaction time was 24 h at 20 bar or 14-15 h at 100 bar. Conversions for all reactions above were > 95 % and the experimental error for % yield and % *ee* were ±10 and ±4, respectively. Catalysts **5**, **8**, and **9** produced (*S*)-**7**. Catalysts **10** produced (*R*)-**7**. Yields are ^{1}H NMR values measured with an internal standard (1,3,5-trimethoxybenzene). Enantiomeric excess determined by HPLC. [b)] 20 or 100 bar H_2 [c)] 20 or 100 bar H_2, 1 eq. DBU added (relative to **6**). [d)] 10 bar $CO_{2(g)}$ added, followed by enough $H_{2(g)}$ to bring the total pressure to 20 or 100 bar. [e)] 10 bar $CO_{2(g)}$ added, followed by enough $H_{2(g)}$ to bring the total pressure to 20 or 100 bar, 1 eq. DBU added (relative to **6**).

Table S2. The effects of reaction time on the conversion and enantioselectivity of the asymmetric hydrogenation of **6** using catalyst **5** in methanol or **8** in IPA.[a]

		3 h	6 h	15 h
Additive	Cat./ solvent	% yield (% *e.e.*)	% yield (% *e.e.*)	% yield (% *e.e.*)
CO_2	**5**/MeOH	58[c] (47)	84 (53)	71 (71)
	**8**/IPA	78 (70)	75 (71)	62 (70)
CO_2 + base [b)]	**5**/MeOH	74[c] (49)	85 (51)	69 (71)
	**8**/IPA	76 (69)	82 (70)	83 (72)

[a)] Experiments were done in triplicate and at RT in a 160 mL stainless steel vessel containing 10 mg **6** and 2 mL methanol in a 1 dram vial, with 10 bar $CO_{2(g)}$ added, followed by enough $H_{2(g)}$ to bring the total pressure to 100 bar. Conversions for all reactions above were ≥ 95 %, unless otherwise stated, and the experimental error for % yield and % *ee* were ±10 and ±4, respectively, except as indicated. Catalysts **5** and **8** produced (*S*)-**7**. Yields are ^{1}H NMR values measured with an internal standard (1,3,5-trimethoxybenzene). Enantiomeric excess was determined by HPLC. [b)] The base used with catalyst **5** was $CyNMe_2$. The base used with catalyst **8** was *i*Pr_2NEt. In both cases, the amount of base was 1 equivalent (relative to **6**) [c)] % Conversion was 81-84 %.

Table S3. The effects of chiral bases, compared to DBU, on the conversion and enantioselectivity of the asymmetric hydrogenation of 2-phenylprop-2-en-1-amine, **6**, in the presence of H_2 and CO_2.[a)]

Additive	Cat.	% yield	% *ee*
CO_2 + DBU	**5**	94	73
	**8**	80	70
	**10**	69	76
CO_2 + (+)-cinchonine	**5**	n/a	49
	**8**	n/a	75
CO_2 + (-)-cinchonidine	**5**	n/a	51
	**8**	n/a	75
CO_2 + (+)-**11**	**5**	84	65
	**8**	90	77
	**10**	94	74
CO_2 + (-)-**11**	**5**	84	64
	**8**	88	76
	**10**	96	71

a) Experiments were done in triplicate and at RT in a 160 mL stainless steel vessel containing 10 mg **6**, 2 mL solvent, and 1 eq. of base (relative to **6**) in a 1 dram vial under 10 bar CO_2 with H_2 then added to bring the total pressure to 100 bar. The reaction was run for 6 h. Catalysts **5** and **10** were in MeOH, while catalyst **8** was in IPA. Conversions for all reactions above were > 95 % and the experimental error for % yield and % *ee* were ±10 and ±4, respectively. Catalysts **5** and **8** produced (*S*)-**7**. Catalyst **10** produced (*R*)-**7**. Yields are ^{1}H NMR values measured with an internal standard (1,3,5-trimethoxybenzene). Yields were not measured with (+)-cinchonine and (-)-cinchonidine. Enantiomeric excess determined by HPLC.

9. References:

[1] W. l. F. Armaego, C. L. Chai, *Purification of Laboratory Chemicals*, Elsevier Ltd, Burlington, MA, **2003**.

[2] T. Ohmura, K. Masuda, I. Takase, M. Suginome, *J. Am. Chem. Soc.* **2009**, *131*, 16624–5.

[3] Z. Ni, L. Giordano, A. Tenaglia, *Chemistry* **2014**, *20*, 11703–6.

[4] C. B. Tripathi, S. Mukherjee, *Angew. Chem. Int. Ed.* **2013**, *52*, 8450–3.

[5] B. Mohan Reddy, V. Venkata Ramana Kumar, N. Chinna Gangi Reddy, S. Mahender Rao, *Chin. Chemi. Lett.* **2014**, *25*, 179–182.

[6] A. Garzan, A. Jaganathan, N. Salehi Marzijarani, R. Yousefi, D. C. Whitehead, J. E. Jackson, B. Borhan, *Chemistry* **2013**, *19*, 9015–21.

[7] Z.-C. Duan, X.-P. Hu, C. Zhang, D.-Y. Wang, S.-B. Yu, Z. Zheng, *J. Org. Chem.* **2009**, *74*,

9191–4.

[8] C. Baumgartner, C. Eberle, F. Diederich, S. Lauw, F. Rohdich, W. Eisenreich, A. Bacher, *Helv. Chim. Acta* **2007**, *90*, 1043–1068.

[9] D. J. Dumas, *J. Org. Chem.* **1988**, *53*, 4650–4653.

[10] D. A. Fort, T. J. Woltering, M. Nettekoven, H. Knust, T. Bach, *Chem. Commun.* **2013**, *49*, 2989–91.

[11] J. P. Holland, P. J. Barnard, S. R. Bayly, H. M. Betts, G. C. Churchill, J. R. Dilworth, R. Edge, J. C. Green, R. Hueting, *Eur. J. Inorg. Chem.* **2008**, *2008*, 1985–1993.

[12] Z. Liang, L. Ju, Y. Xie, L. Huang, Y. Zhang, *Chemistry* **2012**, *18*, 15816–21.
